# Measuring barriers to fistula care: investigating composite measures for targeted fistula programming in Nigeria and Uganda

**DOI:** 10.1186/s12905-021-01288-3

**Published:** 2021-04-07

**Authors:** Pooja Sripad, Elly Arnoff, Charlotte Warren, Vandana Tripathi

**Affiliations:** 1 Reproductive Health Program, Population Council, Washington, DC USA; 2 Independent Consultant, Brooklyn, NY USA; 3 Fistula Care Plus, EngenderHealth, New York, NY USA

**Keywords:** Barriers, Fistula, Care-seeking, Stigma, Social, Awareness, Inaccessibility, Health systems, Measure validation

## Abstract

**Background:**

Accessing surgical repair poses challenges to women living with female genital fistula who experience intersectional vulnerabilities including poverty, gender, stigma and geography. Barriers to fistula care have been described qualitatively in several low- and middle-income countries, but limited effort has been made to quantify these factors. This study aimed to develop and validate composite measures to assess barriers to accessing fistula repair in Nigeria and Uganda.

**Methods:**

This quantitative study built on qualitative findings to content validate composite measures and investigates post-repair client surveys conducted at tertiary hospitals in Northern and Southern Nigeria and Central Uganda asking women about the degree to which a range of barriers affected their access. An iterative scale development approach included exploratory and confirmatory factor analyses of two samples (n = 315 and n = 142, respectively) using STATA 13 software. Reliability, goodness-of-fit, and convergent and predictive validity were assessed.

**Results:**

A preliminary 43-item list demonstrated face and content validity, triangulated with qualitative data collected prior to and concurrently with survey data. The iterative item reduction approach resulted in the validation of a set of composite measures, including two indices and three sub-scales. These include a Financial/Transport Inaccessibility Index (6 items) and a multidimensional Barriers to Fistula Care Index of 17 items comprised of three latent sub-scales: Limited awareness (4 items), Social abandonment (6 items), and Internalized stigma (7 items). Factor analyses resulted in favorable psychometric properties and good reliability across measures (ordinal thetas: 0.70–0.91). Higher levels of barriers to fistula care are associated with a woman living with fistula for longer periods of time, with age and geographic settings as potential confounders.

**Conclusions:**

This set of composite measures that quantitatively captures barriers to fistula care can be used separately or together in research and programming in low- and middle-income countries.

**Supplementary Information:**

The online version contains supplementary material available at 10.1186/s12905-021-01288-3.

## Background

Female genital fistula results in uncontrollable leakage of urine and/or feces, and is often caused by obstructed labor without timely or adequate management. Globally, between 1 and 2 million women are living with fistula and thousands of new cases occur annually [1]. Fistula disproportionally affects poor and marginalized women and girls who lack timely access to quality obstetric care and to follow-up services required in addressing maternal morbidities [2–4]. Policies and programs in low- and middle- income countries (LMICs) inadequately address barriers faced by women living with fistula in accessing treatment services. Women living with fistula frequently experience humiliation, isolation and stigma, among many other barriers, that prevent them from seeking and receiving comprehensive care and treatment, including surgery and follow up support services. Studies indicate that barriers cut across psycho-social, cultural, awareness, social, financial, transportation, facility shortages, quality of care, and political dimensions [5]. The complexity of these barriers and relative rarity of the condition create measurement challenges for countries seeking to monitoring access to fistula care.

Understanding the layered barriers and intersectional vulnerabilities of poverty, gender, stigma, and geography faced by women in need of fistula care is useful in better targeting fistula services and designing programs to reduce obstacles to care seeking and treatment [6]. In sub-Saharan Africa, qualitative studies over the last decade have explored multiple barriers faced by women living with fistula [5, 7–12]. Financial barriers observed across studies include actual and perceived costs of transportation to hospitals and ancillary services during surgery and the post-operative recovery period for women and accompanying family members [8, 10, 13, 14]. Women also face deterrents and delays in care-seeking related to health systems quality (e.g. inadequate antenatal, childbirth and postnatal care and limited health worker knowledge) as well as gaps in individual and community awareness of fistula as a condition, its causes, consequences, and treatment options [15, 16]. Additionally, psychosocial barriers intersect with gender dynamics in the household to leave many women isolated and reinforce a deep sense of shame and low self-worth. These barriers may affect women’s ability to manage self-care in the home, engage in socioeconomic and cultural activities, or decide to seek repair and reintegration services [7, 9, 11, 17, 18]. While barriers to fistula care have been described qualitatively in several LMICs, limited effort has been made to quantitatively and comprehensively measure these factors, in a standardized and comparable way.

Quantitative fistula studies to date primarily focus on prevalence estimation (epidemiology) or profile experiences for clinical care [1, 19–21], with limited attention to measuring the range of barriers to fistula care. These studies have, however, demonstrated aspects of women’s experience with fistula that are conceptually and empirically (cross-sectionally) associated with barriers to seeking, reaching, and receiving fistula care. In Nigeria and Uganda, according to a cross-country survey, the median length of time a woman lives with fistula before seeking care (~ 1 and ~ 2 years, respectively) is likely associated with the nature and magnitude of barriers she faces [20]. A quantitative analysis of Demographic and Health Survey (DHS) data in sub-Saharan Africa, suggests that age and levels of education may have indirect associations with barriers and enablers of care; namely, those with higher education levels may be more likely to attend antenatal care, have better birth preparedness and elevated knowledge of fistula prevention and care options [22]. The sociocultural and health systems contexts of fistula care-seeking are important to consider, including spousal, family, community social support or lack thereof on the one hand and formal and informal care alternatives (e.g. surgical/rehabilitation services and/or traditional healers) on the other [20, 23, 24]. Given the often-hidden nature of the population living with fistula, limited data on the fistula burden in LMICs, and backlog of cases awaiting surgical treatment at hospitals, our current study relies on women who have reached care to make inferences about those who have not.

Composite measures (scales and indices) offer an opportunity to better understand barriers to fistula care and design interventions aimed at reducing obstacles and increasing equity-promoting solutions. While the cross-country DHS study showed seven discrete reasons for not seeking fistula care-ranging from unawareness of fistulas repairability and service locations, to expense and distance, to embarrassment, to limited permission to seek care, and poor quality care– these were not cumulatively analyzed at the level of the woman herself [22]. Composite measures are of growing interest in maternal, newborn, and child health globally, e.g., related to the increased focus on experienced quality of care [25–27]. Scales measure latent concepts like “barriers” and are recognized as acceptable in health program settings that focus on marginalized or stigmatized populations as seen in the HIV and mental health fields [28, 29]. Given the layered, interrelated, yet conceptually congruent nature of the barriers experienced by women with fistula across LMICs countries [5] and documented in Nigeria and Uganda in particular [15, 16], composite scales related to this maternal morbidity may deepen our understanding of a complex psychometric phenomenon and offer measurement tools for wider research and programmatic use.

No composite measure of barriers has been developed to our knowledge in the context of fistula repair. Our study aimed to develop and validate composite measures of barriers to accessing fistula care currently faced by women living with fistula in Nigeria and Uganda. The resulting measures—indices and scales—can be used to inform future planning, programming and resource allocation for fistula care.

## Methods

### Study design and setting

This study applied an iterative approach, guided by scale development principles and item reduction [30], including exploratory and confirmatory factor analyses to develop a quantitative composite measure assessing barriers to fistula care. The study was nested within an evaluation of a complex social behavior change (SBC) intervention to reduce barriers to fistula care in Nigeria and Uganda under the USAID-funded Fistula Care *Plus* Project implemented by EngenderHealth [23].

Implemented among fistula patients at accredited fistula repair centers in Ebonyi in southern Nigeria, Katsina in northern Nigeria, and the Central 1 sub-Region of Uganda, this study validated the composite measure in multiple settings. The fistula centers in Nigeria were located at well-functioning hospitals that provide surgical fistula repair on a routine basis, and treat women with fistula in a timely manner, with limited case backlog. Katsina experienced greater backlog than Ebonyi due to the higher estimated fistula burden in the north of Nigeria. The fistula center in Central 1 sub-Region, Uganda, one of the country’s leading fistula treatment sites, offers mainly pooled/camp-based services for fistula repairs, which generally indicates some level of case backlog at the community level. The estimated number of fistula cases in Ebonyi, Katsina and Central-1 sub-Region, Uganda was 696, 1895, and 758, respectively [31].

### Participant sample and method

The target population for use of these composite measures is any woman living with fistula; however, given the inability to sample this group at the population level, we relied on a study population of women with fistula who reached a care facility after experiencing similar barriers. Our study population comprises women aged 15 years and above who received fistula repair at the accredited fistula centers in Ebonyi and Katsina, Nigeria and Central Uganda. Women were invited to participate post-surgery during their two-week stay at the respective recovery wards in these centers. Trained data collectors approached potential participants at the fistula centers and engaged them in a comprehensive informed consent process. Given the study only interviewed “emancipated minors” –adolescents aged 15–17 years not living under the control of parents or guardians, married, or looking after their own child, no parent or guardian consent was required. After documenting informed consent by signature or thumbprint (for those with limited literacy), data collectors administered a post-repair client survey in the local language (Igbo and Hausa in Nigeria, and Luganda in Uganda). Post-repair client surveys were conducted at baseline and endline of the larger SBC intervention to develop and validate the composite measures. Surveys were paper-based in Nigeria and collected on tablets using Open Data Kit software in Uganda.

### Measurement

Post-repair client surveys explored women’s sociodemographic characteristics, reproductive health and fistula history, and the barriers and enablers to fistula care they experienced (Additional File 1). The thematic categories of barriers and range of items that formed the composite measure were developed iteratively based on a literature review [5], and qualitative exploration of local barrier constructs [15, 16]. Similarly identified qualitative barriers reported across the study settings—through focus groups and in-depth interviews—allowed researchers to develop a harmonized set of items for Nigeria and Uganda. We assessed 43 items or barriers to fistula care that captured awareness of the condition’s causes, manifestations, and care options, restrictive cultural beliefs, gender norms reflecting women’s care-seeking ability, psychological consequences, social stigma, financial costs, transport and infrastructure, health care quality and interactions. Each item asked the degree to which a barrier item affected a woman’s access to fistula repair and was scored on a 4-point Likert scale with increasing options from ‘strongly disagree’, to ‘disagree’, to ‘agree’, to ‘strongly agree.’ In Uganda, the scale was administered as a 5-point Likert with a ‘neutral’ category.

Items were translated into Igbo, Hausa, and Luganda and back translated into English to check for meaning and comprehension. During the baseline assessment, the items were pretested with a several women in each fistula center prior to the study start to refine wording and understand the relevance of the full list of barriers. An open-ended section at the end of the post-repair survey asked about whether any additional barriers had been experienced by the participant, or whether any included barriers were deemed irrelevant. Fistula clients’ responses suggested the study team should retain all the items across the three sites at the start of the measure development process.

### Analysis

Given the varied domains of barriers to fistula treatment identified in the literature review and formative research, we began with the assumption that this is a multidimensional construct. We applied an iterative process of item reduction and exploratory and confirmatory factor analysis using the baseline and endline samples, respectively. We reverse-coded positively worded items to create consistent response categories for barriers; each barrier was scored between 1 and 4 (‘strongly disagree’ to ‘strongly agree’). Neutral responses, which comprised less than 10% of the responses across items in Uganda were re-classified as “agree,” one of the mid-point categories, to ensure comparability of data across settings and retain statistical power. We explored distributions of all the items to assess uniformity in response categories for the psychometric analyses. We generated scale scores and evaluated the distribution of the full scale and sub-scales. All statistical analysis were conducted using Stata 13 software.

To determine the factor structure and assess construct validity, we conducted exploratory factor analyses (EFA) on the baseline sample to identify the potential number of factors that fit the data. The EFA specifications were selected based on an assessment of normality in the response distributions as well as of correlation among the potential factors. Following recommended criteria [30], we conducted a principal component analysis (PCA), applying parallel analysis and scree plot review to identify the number of factors. We retained the number of factors with eigenvalues over 1.00. Within this plausible factor structure, we assigned items to factors based on face and content validity considerations through a consultative process and by exploring inter-item correlation matrices.

We did this in combination with an item reduction approach, removing items with extremely low (< 10%) and high (> 90%) frequencies that approached floor and ceiling effects, to ensure response variability. Items were removed if they had high proportions of missing data. We tested the normality of item responses to determine the type of factor analysis to implement. Items with low uniqueness (< 0.7), low factor loadings (< 0.3), and interpretability based on face and content validity were retained for the confirmatory factor analysis (CFA). We compiled the items excluded through this process but with high face and content validity into a separate index.

We conducted a CFA using endline sample data, to test the emergent factor structure from the EFA and retained items with statistically significant factor loadings (*p* < 0.05). Statistical tests measuring model adequacy were conducted and interpreted based on common criteria including the root mean square error of approximation (RMSEA), comparative fit index (CFI), Tucker-Lewis Index (TLI), and the standardized root mean square residual (SRMR). Modification indices were built into the CFA’s structural equation modeling to adjust for similarly worded items and assess goodness-of-fit. We also assessed scale invariance across sites to account for any sample differences between the distinct geographies of Ebonyi, Katsina, and Uganda.

Scale and index reliability and validity were assessed using standard correlation tests and related criteria. Reliability, or internal consistency of items, was assessed using Cronbach’s alpha and ordinal theta. The latter assumes polychoric correlation, useful when items have limited response categories (four in the case of assessed items). A standard reliability cutoff of 0.7 was adopted to assess adequate internal consistency, i.e., how well the items relate to one another to capture the overall construct of barriers to fistula care. We assessed convergent validity by exploring associations among scores with the theoretically correlated sub-scales and indices resulting from these measure development procedures. Predictive validity was assessed by exploring associations of the indices and sub-scales with variables that are theoretically expected to be correlated: the length of time a woman has been living with fistula and whether she has sought treatment in the past. Bivariate and multivariable predictive validity models were assessed, controlling for age, education level, and fistula repair center.

## Results

### Demographics and fistula history

A total of 457 women participated in the post-repair client survey, including 315 at baseline (EFA sample) and 142 at endline (CFA sample). Across sites, our EFA and CFA samples of fistula clients who had received repair resided predominantly in rural areas, were between 15 and 45 years of age with a majority in the 26–35-year age group, and were majority Christian (Table 1). Education was low in both samples with nearly half of women having a primary school education (40–44%), between 20 and 33% with no education, 15–21% with secondary education, and 5–15% with higher than secondary. Across samples, the majority of women were married or cohabiting and 43–47% of women reported currently working for pay, while a similar range reported not working for an income in in the last year. Most women developed fistula after childbirth (obstetric) and about half the women in both samples sought treatment for their leaking previously.

**Table 1:** Socio-demographic characteristics of EFA and CFA samples

	Exploratory, n = 315		Confirmatory, n = 142	
	n	%	n	%
Site				
Ebonyi, Nigeria	91	28.9	51	35.9
Katsina, Nigeria	81	25.7	44	31.0
Uganda	143	45.4	47	33.1
Age				
15–25 years	102	32.4	41	28.9
26–35 years	89	28.3	62	43.7
36–45 years	60	19.0	17	12.0
46–55 years	7	2.2	12	8.5
56+ years	10	3.2	3	2.1
Education				
None	63	20.0	47	33.1
Primary	129	41.0	62	43.7
Secondary	66	21.0	21	14.8
More than secondary	17	5.4	5	3.5
Only Quranic	39	12.4	7	4.9
Currently working	137	43.5	67	47.2
Marital status				
Single (never married)	30	9.5	10	7.0
Cohabitating	22	7.0	34	23.9
Married	193	61.3	76	53.5
Divorced/separated	56	17.8	10	7.0
Widowed	13	4.1	9	6.3
Number of living children				
None	130	41.3	35	24.6
1–2	93	29.5	46	32.4
3–4	61	19.4	36	25.4
5 or more	50	15.9	19	13.4
Last pregnancy delivered at:				
Hospital/facility/PHC	272	86.3	106	74.6
Home	23	7.3	17	12.0
Home with TBA	7	2.2	14	9.9
Prolonged labor during last delivery	223	70.8	91	64.1
Delivery outcome				
Live baby	99	31.4	50	35.2
Live baby but dies few hours later	39	12.4	23	16.2
Stillbirth	173	54.9	64	45.1
Length of time living with fistula				
< 1 year	176	55.9	69	48.6
1–2 years	35	11.1	24	16.9
3–4 years	50	15.9	14	9.9
5–10 years	18	5.7	13	9.2
10+ years	32	10.2	19	13.4
Problem of leaking started				
After delivering a live/stillborn baby	271	86.0	133	93.7
After abdominal/pelvic surgery	32	10.2	2	1.4
After a sexual assault or other injury	2	0.6	4	2.8
Sought treatment for leaking	142	45.1	70	49.3

### Factor analysis and item reduction

After reviewing distributions of the 43 items, the PCA and associated analyses identified between three and five factors that fit the data. We assigned items within the plausible factor structure based on the procedures described above (Additional file 2: Supplemental Table 1). The item reduction process resulted in the removal of five items with extremely low (< 10%) and high (> 90%) frequencies. One item was removed due to a high proportion of missing responses and another was removed for demonstrating poor correlation across all the potential factors. As our responses were not normally distributed, we conducted EFA of the 36 items with the iterative principal factor specification. Similarly, we conducted EFA with promax rotation as our factors were correlated.

Within the EFA, fifteen items were removed due to high uniqueness (> 0.7), which indicates that these items did not effectively relate to any one of the factors, full scale, or sub-scales. Two items were removed for low (< 0.3) and mixed (multiple factor) loadings in the EFA. The EFA results suggested that removed items function as independent elements that may affect or moderate the underlying latent barriers to care, rather than comprise elements of the composite measure. The EFA identified a three-factor solution, which we conceptualized as three sub-scales reflecting latent barriers to fistula care: limited awareness, social abandonment, and internalized stigma. Six of removed items were compiled into a separate Financial/Transport Inaccessibility Index (Table 2) given their prevalence, distribution and relevance as distinct factors that relate to access.

**Table 2:** Financial/transport inaccessibility index

Item
I did not have money to pay for medical care to treat my fistula
I was unable to work because of stigma associated with my fistula condition
There are not enough transport options to get to the fistula center
The cost of transportation to repair sites and accommodation was too high
The repair facility was too far
The road conditions were bad

We conducted a CFA of each of the sub-scales and then the full scale, comprised of 19 items, to test the emergent three-factor structure. Two items were removed due to low factor loadings in the CFA and poor reliability statistics. While all three sub-scales converged, the hierarchical multi-factor model did not. Therefore, we cannot conceptualize the final composite measure as a scale. Rather, we present it as a multidimensional Barriers to Fistula Care Index of 17 items with three sub-scales: Limited awareness (four items), Social abandonment (six items), and Internalized stigma (seven items) (Table 3).

**Table 3:** Barriers to fistula care index: sub-scale EFA and CFA factor loadings

Sub-scale domain	Item	EFA factor loadings	CFA factor loadings
Limited awareness	I did not know that fistula is a medical condition that can be treated	0.61	0.77
	I believed that having fistula was a curse	0.47	0.54
	I believed that my fistula was caused by diabolic means	0.62	0.28
	I did not know where to go for fistula repair	0.63	0.54
Social abandonment	People who knew I had fistula avoided me	0.39	0.30
	My husband/intimate partner treated me poorly initially	0.90	0.93
	My husband/intimate partner treated me poorly later on	0.93	0.92
	My husband/intimate partner abandoned me	0.86	0.95
	I did not have someone to care for me and help me manage my condition at home	0.49	0.54
	I did not have someone to support me in seeking and reaching care at the fistula center	0.36	0.43
Internalized stigma	I felt ashamed of having fistula	0.63	0.69
	I felt worthless	0.63	0.56
	I felt guilty because I had fistula	0.62	0.44
	I felt I am not as complete as a person because I had fistula	0.74	0.69
	Having fistula made me feel unclean	0.67	0.46
	I felt embarrassed because of my condition	0.57	0.52
	I felt isolated because of my fistula condition	0.40	0.43

Model adequacy assessment during CFA revealed the need for modification indices for each of the sub-scales. As such we adjusted for covariance in the error terms of the items. Goodness of fit statistics (Table 4) across the structural equation models were acceptable, with RMSEAs < 0.1, CFIs and TLIs > 0.9 (in some cases > 0.95), and SRMR < 0.01 (and in some cases < 0.05). While we were unable to conduct tests of group invariance to ensure fit across sites due to low sample sizes in each site, we attempted an alternative check to ensure that factor loadings/structure did not differ appreciably between study sites. We evaluated our CFA models, controlling for site, and found that factor loadings for Limited awareness and Social abandonment were similar to uncontrolled models with variable statistical significance (significant for Limited awareness), but did not converge for Internalized stigma.

**Table 4:** Model-fit statistics—CFA

	RMSEA	CFI	TLI	SRMR
Sub-scales				
Limited awareness (4 items)	0.097	0.989	0.931	0.024
Social abandonment (6 items)	0.067	0.992	0.985	0.049
Internalized stigma (7 items)	0.075	0.945	0.912	0.051

*RMSEA* root mean squared error of approximation, *CFI* comparative fit index, *TLI* Tucker–Lewis Index, *SRMR *standardized root mean square residual

### Reliability and validity

We generated summary scores of the indices and sub-scales and evaluated their distributions (Table 5). The distributions of the 17-item multidimensional Barriers to Fistula Care Index (score range: 17–68) and its six-item Limited awareness sub-scale (score range: 4–16), six-item Social abandonment sub-scale (score range: 6–24), and seven-item Internalized stigma sub-scale were fairly consistent across EFA and CFA samples. The six-item Financial/Transport Inaccessibility Index (score range 6–24) was also similarly distributed across samples. We observed some variation in distributions of the summary scores across the three sites in the EFA and CFA samples.

**Table 5:** Distribution and reliability of indices and sub-scales

Overall distribution and reliability														
	Exploratory							Confirmatory						
	Mean	SD	Possible range			Alpha	Ordinal theta	Mean	SD	Possible range			Alpha	Ordinal theta
Total barrier index (17 items)	45.80	9.59	17	–	68	0.85	0.88	49.21	9.01	17	–	68	0.85	0.89
Sub-scales														
Limited awareness (4 items)	10.29	3.37	4	–	16	0.71	0.77	10.54	3.29	4	–	16	0.69	0.74
Social abandonment (6 items)	12.89	4.99	6	–	24	0.84	0.86	10.54	3.29	6	–	24	0.86	0.88
Internalized stigma (7 items)	22.45	4.51	7	–	28	0.82	0.91	11.82	4.33	7	–	28	0.73	0.85
Financial/transport inaccessibility index (6 items)	16.24	2.99	6	–	24	0.62	0.70	16.99	2.96	6	–	24	0.67	0.75

Site-specific distribution												
	Ebonyi, Nigeria				Katsina, Nigeria				Uganda			
	EFA		CFA		EFA		CFA		EFA		CFA	
	Mean	SD	Mean	SD	Mean	SD	Mean	SD	Mean	SD	Mean	SD
Total barrier index (17 items)	43.22	7.81	50.27	9.44	40.99	5.85	41.03	4.03	49.88	10.42	55.02	6.12
Sub-scales												
Limited awareness (4 items)	9.86	2.46	10.98	3.09	8.05	1.95	7.42	1.80	11.83	3.69	12.91	2.15
Social abandonment (6 items)	12.31	4.41	11.82	5.84	13.38	3.04	10.13	1.79	12.58	6.06	13.26	3.37
Internalized stigma (7 items)	20.63	4.06	27.47	3.65	19.66	3.39	23.56	3.06	25.09	3.79	28.85	2.81
Financial/transport inaccessibility index (6 items)	16.54	3.03	18.08	3.64	14.95	1.61	14.88	1.35	16.81	3.34	17.66	2.13

The index and sub-scale reliability or internal consistency, assessed using Cronbach’s alpha and ordinal theta, were similar and high in both EFA and CFA samples, all above the 0.7 threshold (Table 5). The multidimensional Barriers to Fistula Care Index exhibited a cross-sample internal consistency of 0.85–0.89, which suggests that the items, together, relate to one another one and capture the overall construct of barriers to fistula care. The sub-scales of Limited awareness, Social abandonment, and Internalized stigma demonstrated moderate to high internal consistency (0.69–0.77; 0.86–0.88; 0.73–0.91, respectively). The Financial/Transport Inaccessibility Index was moderate (0.62–0.75). Cross-site index distributions suggest that the highest barrier index scores in Uganda followed by Ebonyi and Katsina; all three sites show similar financial/transport inaccessibility. Sub-scales indicate similar levels of social abandonment and internalized stigma across all three sites, with higher awareness barriers in Uganda.

We assessed convergent validity by exploring associations of the full multidimensional Barriers to Fistula Care Index scores with scores on each of the sub-scales and the Financial/Transport Inaccessibility Index (Table 6). Though variable in magnitude, all sub-scale scores were positively and significantly associated with the Barriers to Fistula Care Index score and each other, indicating convergent validity of the multiple barrier dimensions. The Barriers to Fistula Care Index and sub-scale scores were also associated with the separate Financial/Transport Inaccessibility Index score, further supporting convergent validity of latent and other barriers.

**Table 6:** Associations between indices and sub-scales (convergent validity)

	Exploratory				Confirmatory			
	Financial/transport inaccessibility	Limited awareness	Social abandonment	Internalized stigma	Financial/transport inaccessibility	Limited awareness	Social Abandonment	Internalized stigma
Barriers to Fistula Care Index	1.66***	2.00***	1.43***	1.67***	1.72***	2.21***	1.60***	1.82***
Sub-scales								
Limited awareness	0.43***				0.45***			
Social abandonment	0.58***	0.18***			0.61***	0.31***		
Internalized stigma	0.64***	0.35***	0.32***		0.65***	0.47***	0/.6***	

All values presented are beta coefficients

*p < 0.05; **p < 0.01; ***p < 0.001

Predictive validity was assessed by exploring associations of the Barriers to Fistula Care Index, sub-scales (Limited awareness, Social abandonment, and Internalized stigma), and the Financial/Transport Inaccessibility Index with theoretically correlated variables: the length of time a woman has been living with fistula and whether she ever sought treatment in the past (Table 7). We additionally tested multivariable models controlling for age, education level, and site, which demonstrated similar results, although the effect sizes were smaller and statistical significance varied because of the small sample and model sizes. As expected, there were more robust associations between Index and sub-scale scores and years living with fistula—the greater the barriers and inaccessibility, the longer women live with the condition—relative to seeking treatment previously. Multivariable regressions showed that only age seemed to be a significant confounding factor for the Barriers to Fistula Care index sub-scales; while site appeared be a confounder only in the CFA for the Social abandonment sub-scale and EFA in the Internalized stigma sub-scale.

**Table 7:** Associations of indices and sub-scales with related outcomes (predictive validity)

	Years living with fistula (linear outcome)		Seeking prior treatment (binary outcome)	
	Exploratory	Confirmatory	Exploratory	Confirmatory
Barriers to Fistula Care Index (17 items)	0.16***	0.16**	0.03**	0.02
Sub-scales				
Limited awareness (4 items)	0.44***	0.30^†^	0.10**	0.06
Social abandonment (6 items)	0.20**	0.32**	0.04	0.04
Internalized stigma (7 items)	0.23**	0.20	0.06*	0.00
Financial/transport inaccessibility index (6 items)	0.17	0.20	0.01	− 0.03

All values presented are beta coefficients

*p < 0.05; **p < 0.01; ***p < 0.001; ^†^0.1

## Discussion

To our knowledge, this is one of the first studies to develop a set of composite measures that quantitatively captures barriers to fistula care (Additional file 2: Supplemental Table 2). The multidimensional Barriers to Fistula Care Index comprises a series of latent sub-scales—Limited awareness, Social abandonment, and Internalized stigma—that can be used separately or together in research and program settings. The Financial/Transport Inaccessibility Index is conceptually distinct from the three sub-scales and overall Barriers to Fistula Care Index (Additional file 2: Supplemental Table 2). Composite measures demonstrate favorable psychometric properties, demonstrate functionality across settings, and are associated with years living with fistula and prior treatment. While the Barriers to Fistula Care Index and subscales primarily assess reasons why women are unable to obtain care and mostly relate to the ‘first delay’ in care-seeking, the Financial/Transport Inaccessibility Index speaks primarily to the ‘second delay’. The ‘third delay’ that relates to quality of health care was incongruent both conceptually and psychometrically given the data in our samples. This may be due to the rarity of the condition or to women’s limited exposure to and perceptions about relevant health services.

There are some limitations to the analysis presented here. We lacked power to sufficiently assess sub-scale stability across geographic areas. As this work was nested within a larger body of work and given the challenge of recruiting repaired women, let alone women still living with fistula, we were unable to draw a large enough sample in each individual site. Future studies should test for invariance across settings, including ensuring adequate sample size in each setting. Another limitation was the fact that our sample of women were post-repair; we have assumed similarity among the barriers faced by women who eventually reached fistula care and those women who have not yet done so. Ideally, barriers among women living with fistula who have not yet sought, reached, or received care would also be assessed to validate this assumption and adjust for any selection bias. However, in practice, this population is very difficult to identify and sample in a representative manner.

The sub-scales or dimensions of the Barriers to Fistula Care Index align with much of the qualitative literature regarding the experience of living with fistula and resonates with quantitative DHS analyses on treatment seeking in sub-Saharan Africa [22]. Our study shows similar types and levels of barriers—including limited awareness, internalized stigma, social abandonment. It confirms that internalized stigma (e.g., that one is dirty) and being avoided or neglected by spouses, family and society (‘social abandonment’) as an commonly ascribed reasons for delayed care-seeking in sub-Saharan Africa [5]. For example, a qualitative study found that obstetric fistula survivors in Addis Ababa coped with their emotions through suicidal thoughts, avoiding family and community members, and concealing their story and circumstances [9]. As such, this sense of shame and compounded barriers often leads to women resorting to different coping mechanisms including but not limited to managing the condition at home, drawing limited social support mechanisms, seeking religious or spiritual guidance, and alternative care [8, 10, 11, 17, 18]. While all three sub-scales measures barriers to care, the Internationalized stigma and Social abandonment sub-scales may be useful to further adapt in understanding care seeking for post-repair and reintegration services [32].

Given the hard-to-reach nature and multiple vulnerabilities of and constraints faced by women living with fistula, the composite barrier measures can be used to inform future programming and resource allocation for fistula in a particular country context. As LMICs vary substantially in the way fistula surgeries, counselling, and reintegration services are structured, these composite measures can be applied at the population level to select optimal outreach, screening, and service delivery models. Quantifying barriers may help elucidate what is needed at the community level to promote access to care. The relative scores on sub-scales can influence targeting outreach, case identification, and referral. The indices and scales have implications beyond fistula strategies and programming; they may guide a better understanding of care-seeking for a wider range of reproductive and maternal morbidities as well as stigmatized conditions. For example, service design and delivery for pelvic organ prolapse and other health areas that require surgical intervention or continuous care (e.g. HIV, TB) could benefit by adapting the measures presented here [33].

We recommend further research replicating these measures elsewhere as well as increasing sample sizes to assess sub-groups of women living with fistula by age and geography. As these indices and scales provide a lens into community-relevant barriers, we encourage the composite measures be tested further through implementation research and large-scale program and health systems monitoring to improve access for the most vulnerable groups. Despite some variation in the summary composite scores, in Nigeria and Uganda, we recommend targeting behavioral interventions toward communities that destigmatize fistula and promote inclusion (non-abandonment) of women living with and recovering from fistula repairs, alongside innovations to enhance awareness, finance and transportation barriers to fistula care.

## Conclusion

This is one of the first studies to develop composite measures quantifying barriers to fistula care by assessing reliability and validity in a vulnerable population from three distinct sociocultural contexts, northern and southern Nigeria and central Uganda. The reliability and validity of the multidimensional Barriers to Fistula Care Index, associated sub-scales (Limited awareness, Social abandonment, and Internalized stigma), and the Financial/Transport Inaccessibility Index introduce options for integrating measurement and monitoring barriers to care into fistula programs. The psychometric properties of each construct suggest cognitive differences in how women perceive and act on their fistula condition. As such, programs may benefit from contextualized behavioral approaches that target distinct barriers as health and social systems evolve over time addressing the varied challenges of women living with fistula.

## Supplementary Information

**Additional file 1.** Post-Repair Fistula Client Survey Tools.

**Additional file 2: Supplemental Table 1.** Distribution of barrier items. **Supplemental Table 2.** Quantitative measures summary.

## Data Availability

The datasets generated and/or analyzed during the current study are not publicly available, since the data are qualitative and contains respondent identities. Data are available from Population Council upon reasonable request.

## Abbreviations

CFA: Confirmatory factor analysis
EFA: Exploratory factor analysis
LMIC: Low- and middle- income country
PCA: Principal components analysis
