# Supplementary material for: Measuring barriers to fistula care: investigating composite measures for targeted fistula programming in Nigeria and Uganda
Source: BMC Womens Health. 2021 Apr 7;21:142. doi: 10.1186/s12905-021-01288-3 (PMC8028827; doi:10.1186/s12905-021-01288-3)
Supplement: Supplementary file 1 — Additional file 1. Post-Repair Fistula Client Survey Tools. [file 12905_2021_1288_MOESM1_ESM.pdf]

Additional File 1

For "Measuring barriers to fistula care: investigating composite measures for targeted fistula programming in Nigeria and Uganda" Manuscript

Post-Repair Fistula Client Survey Tools

Women's Interview

Fistula Care *Plus* – Baseline - Katsina

Women's post repair interview/barrier checklist (administered during stay at fistula centre)

QUESTIONNAIRE IDENTIFICATION INFORMATION

Questionnaire Number*:*

INTERVIEWER: CODE [____|____] NAME____________________________
DATE OF INTERVIEW: __/____ /_____
TIME INTERVIEW STARTED: HOUR/MINUTES: |___|___| (USE 24 HOURS CLOCK)
SUPERVISOR'S REVIEW: (NAME) ____________________
Signature after editing _________________ DATE ___________________
COUNTRY: Nigeria (01) Uganda (02) CODE |___|___|
STATE: Ebonyi (01) Katsina (02) **CODE |___|___|**

Local Governmental Area (LGA): ____________________________________

Venue of Interview________________________________ (Write name of facility or Division & Location)

Informed Consent Form signed Yes - 1 No - 2
Language used to conduct interview: 1-English, 2- Igbo, 3– Hausa, 4—Combined 5—Other (specify)

____

Interview result: Completed – 1 Postponed – 2 Refused – 3 Incomplete---4

If incomplete (reason____)

Under formatting review

	**SECTION 1: SOCIO DEMOGRAPHIC CHARACTERISTICS**<br>First I would like to ask some questions about you and your household.<br>*Na farko ina son in tambaye wasu tambayoyi game da kai da iyalinka.*	
**101a**	From the time you were born till you were 12 years, did you live in town or in the rural areas?<br><br>*Daga lokacin da aka haife ki har zuwa shekara sha biyu, a ina kike zaune?*<br><br>[READ OUT OPTIONS]	District town………..1<br>Other city or town…..2<br>Rural areas ………….3<br>Outside Katsina ……...4
**101b**	Where are you living now?<br><br>*A ina kike zaune yanzu?*<br><br>[READ OUT OPTIONS]	District town………..1<br>Other city or town…..2<br>Rural areas ………….3<br>Outside Katsina……...4
**102**	How long have you been living in that area (before coming to fistula center)?<br><br>*Tun yaushe kike zaune a can kafin kizo cibiyar?*<br><br>[WRITE IN YEARS OR CIRCLE]	Years ……………………………..1<br>Specify years: ____________<br>Always…………………………….2<br>Visiting ……………………………3
**103**	In what MONTH and YEAR were you born?<br><br>*A wane wata da shekara aka haife ki?*<br><br>[Use events to remember, if needed]	**MONTH** [ ]<br>know month ….. 98<br>**YEAR** [ ]<br>know year……… 8
**104**	How old were you at your last birthday?<br><br>*Shekarar ki nawa a lokacin da kikayi bikin haihuwarki?*<br><br>[WRITE IN. PLEASE RECONCILE IF THERE IS A DIFFERENCE BETWEEN q103 AND q104]	**Years** [ ]<br><br>Don't know……………………..98
**105**	Have you ever attended any school?<br><br>*Kin taba yin makaranta?*	Yes…………1<br>No…………..2---------**Skip to Q109**

**106**	What is the highest level of school you attended: Primary, secondary, or more than secondary?<br><br>*Yaya zurfin iliminki yake?*	Primary .... 1<br>Secondary .... 2<br>More than secondary .... 3<br>Only Quranic……………………….4
**107**	What is the highest you completed at that level?<br>*Wace kololuwar kika kai?*<br><br>[WRITE IN]	Standard/form/class ___________<br>Don't know ……………………….99
**109**	What is your religion?<br><br>*Wane addini kike?*	Catholic………………………………1<br>Other Christian……………………….2<br>Islam…………………………………3<br>Traditionalist…………………………4<br>No religion …………………………..5<br>Other (Specify)_________________....6
**110**	What is your ethnic group?<br><br>*Wace Kabila ce ke?*	Hausa…………………………..1<br>Igbo…………………………....2<br>Yoruba……………………..….3<br>Fulani………………………..…4<br>Ibibio…………………………..…5<br>Ijaw……………………..……...6<br>Kanuri………………................7<br>Tiv……………………………....8<br>Other (specify_____________)….9
**111**	Aside from your normal housework are you currently working?<br><br>*Banda ayyukan ki na gida, kina wani aiki yanzu?*	Yes…….1 **Skip to 113**<br><br>No……..2 **Skip to 112**
**112**	Aside from your normal housework, have you worked for income/pay in the last 12 months?<br><br>*Banda ayyukan ki na gida, kin taba yin aikin da ake biyanki a shekara daya data wuce?*	Yes………1 **Skip to 113**<br><br>No……….2 **Skip to 117**

**113**	What is your occupation, that is, the kind of work you normally do?<br><br>*Wane aiki kika fi yi ako yaushe?*<br><br>[WRITE IN]	Write in:
**114**	What is your monthly income?<br><br>*Kamar nawa kike samu a wata?*<br><br>[IF DON'T KNOW, READ OUT]	Below NGN 5000…………….1<br>NGN 5000-10,000……………2<br>NGN 10,000-15,000 …………3<br>NGN 15,000-20,000…………4<br>NGN 20,000-25,000…………5<br>NGN 25,000-30,000…………6<br>NGN 30,000-35,000…………7<br>NGN 35,000-40,000…………8<br>NGN 40,000 and above………9<br>Don't know……………………98
**115**	Who mainly decides how the money you yourself earn is spent?<br><br>*Wake da alhakin gaya maki yadda zaki kashe kudin da kike samu?*<br><br>[READ OUT]	Self………………………………..1<br>Husband/partner…………………..2<br>Self and husband/partner jointly….3<br>Someone else……………………..4<br>Self and someone else jointly ……5<br>N/A………………………………99
**116**	On average how much of your household's expenditure do your earnings pay for?<br><br>*A kalla kamar nawa kike kashewa a kudin da kike samu a wata?*<br><br>[READ OUT]	Almost none…………………1<br>Less than half………………..2<br>About half…………………….3<br>More than half………………..4<br>All…………………………….5<br>None her income is saved…….6
**117**	What is your marital status?<br><br>*Mine ne matsayin ki a aure?*	Single (Never Married)……1 →**skip to 201**<br>Cohabiting…………………2→**skip to 118**<br>Married…………………….3 →**skip to 118**<br>Separated…………………..4 →**skip to 119**<br>Divorced…………………...5→**skip to 119**<br>Widowed…………………..6→**skip to 119**

**118**	Is your husband/partner living with you now or is he staying elsewhere?<br><br>*Kina zaune da mijinki ko abokin zamanki yanzu? Koko yana wani wuri daban?*	Living with her ……1 Staying elsewhere….2
**119**	Does/did your husband/partner have any other wives beside yourself?<br><br>*Mijinki yana da wasu mata bayan ke?*	Yes…………..1<br>No……………2 --------**Skip to 121**<br>Don't know….98
**120**	Are/were you the first wife or second or.....?<br><br>*Kece ta farko ko tanawa.....?*	Only wife…………1<br>First wife …………2<br>Second wife……….3<br>Third wife…………4<br>Fourth wife ……….5<br>Don't know………..98
**121**	When you first got married or lived with a man was it your choice, or did your family arrange it?<br>*Lokacin da aka yi maki aure da mijinki, shin son ranki ne ko kuwa iyaye suka hada?*	Own choice…………….1<br>Arranged by family ……2
**122**	When you first got married or lived with a man: was the man younger, about the same age or older than you?<br><br>*Mijin ki na farko saurayine ko sa'ankine ko ya girme ki?*<br><br>[PROBE: IF OLDER, HOW MUCH OLDER THAN YOU, LESS THAN 10 YEARS OR MORE THAN 10 YEARS OLDER?]	Younger……………………………1<br>About the same age…………………2<br>Older – less than 10 years older…….3 Older - more than 10 years older ….4<br>Don't know ………………………..98
**123**	How old were <u>you</u> when you first got married or started living with a man?<br><br>*Shekarun ki nawa lokacin da kikayi aure ko kika fara zama da namiji?*	Age in years [ ][ ]<br><br>Can't remember……………..98

**124**	Did your husband ever attend school?<br><br>*Mijinki ya taba yin makaranta?*	Yes……………..1<br>No………………2---------**skip to 201**<br>Don't know…….98----------**skip to 201**
**125**	What was the education level of your husband?<br><br>*Minene zurfin ilimin mijinki?*<br><br>[READ OUT]	Primary……………………....1<br>Secondary……………………2<br>More than secondary………..3<br>Quranic only…………………4<br>Don't know…...……………98 →**skip to 127**
**126**	What is the highest your husband completed at that level?<br><br>*A wane matakin ilimin mijinki yakai?*<br><br>[WRITE IN]	Standard/form/class ________<br><br>Don't know………………..98
**127**	What is the current occupation of your husband, that is, what kind of work does he mainly do?<br><br>*Minene sana'ar mijinki a yanzu?*<br><br>[WRITE IN]	Write in:

SECTION 2: REPRODUCTIVE AND MATERNAL HISTORY

Now I would like to ask you about pregnancy, childbirth, and also some questions about sexual activity and reproductive health in order to better understand some family life issues.

Sashe na Biyu: Tarihin haihuwa da Matanci

Yanzu zan tambayeki akan daukar ciki, haihuwa, Jima'I da yanayin lafiyar iyali.

**201**	How old were you when you first had sexual intercourse?<br><br>*Kina da shekaru nawa kika fara saduwa da namiji?*<br><br>[PROBE: WHAT WAS TYPICAL AGE FOR GIRLS TO GET MARRIED]	10-14 years………………………1<br>15-19 years……………………….2<br>20-24 years……………………….3<br>25 years and above………………4

Under formatting review

**202**	How old were you when you became pregnant the first time?<br><br>*Kina da shekaru nawa kika fara daukar ciki?*	10-14 years……………………….1<br>15-19 years……………………….2<br>20-24 years……………………….3<br>25 years and above………………4
**203**	At the first time you became pregnant, did you want to be pregnant then, did you want to wait until later or did you not want to have the baby at all?<br><br>*Lokacin da kika samu cikin farko kinso hakan, ko kuwa kinso ki saurara sai wani lokaci, ko kuwa a'a gaba daya baki son haihuwar?*	Wanted it then…………………1<br>Wait till later…………………...2<br>Did not want …………………...3
**204**	How many pregnancies have you had? Including those that miscarried?<br><br>*Sau nawa kika samu ciki har da bari?*<br><br>[WRITE IN NUMBER]	Write in:
**205**	How many living children do you have now? (biological children only)<br><br>*Yara nawa gare ki yanzu a raye?*	Write in:
**206a**	During your last (full-term) pregnancy, did you attend antenatal care?<br><br>*Lokacin da kika samu ciki na karshe, kin rinka zuwa awo?*	Yes………..1<br>No…………2**----------skip to 207a**
**206b**	How many times did you attend antenatal care?<br><br>*Saunawa kika je awo?*	Once ………………..1<br>Twice ……………….2<br>Three times………….3<br>Four times …………..4<br>Five or more………....5

**207a**	Where did you deliver?<br><br>*A ina kika haihu?*<br><br>[READ OUT]	At hospital/facility/PHC……….1<br>At home………………………..2<br>At home with TBA……………..3
**207b**	Did you suffer any complications?<br><br>*Kin samu wasu matsaloli wajen haihuwar?*<br><br>[READ OUT, CIRCLE ALL THAT APPLY]	Bleeding…………………………1<br>Prolonged labour………………...2<br>Fitting……………………………3<br>Sepsis …………………………...4<br>Other ……………………………5<br>(Specify_________________)<br>None …………………………….6
**207c**	What was your delivery outcome?<br><br>*Meye sakamakon haihuwar?*<br><br>[READ OUT OPTIONS]	Live baby…………………………………1<br>Live baby but died few hours days later….2<br>Stillbirth…………………………………..3

SECTION 3: FISTULA<br>Now I'm going to ask you a few questions about the development of your fistula.<br><br>***Sashe na ukku: Yoyon Fitsari***<br>*Yanzu zan yi maki yan Tambayoyi game da afkuwar yoyan fitsari.*		
**301**	How long had you been living with fistula?<br><br>*Tun yaushe kike fama da wannan lalurar?*<br><br>[WRITE IN]	Write in:
**302**	When did this problem (LEAKAGE OF URINE AND/OR FECES) start?<br><br>*Yaushe wannan lalura ta Yoyon fitsari/ko Bayan gida ta fara?*<br><br>[LET WOMAN RESPOND; IF SHE IS SILENT, READ OUT]	After you delivered a live or stillborn baby………………………..1<br>After abdominal or pelvic surgery while you were not pregnant….2 → **skip to 304**<br>After a sexual assault, attack, or other injury……………………...3 →**skip to 304**<br>None of the above ………………………………………………4→**skip to 304**<br>Don't know……………………………………………………..99 →**skip to 304**
**303**	Did this delivery (after which leaking started) happen normally, did they pull the baby out, or did they cut you/do an operation?<br><br>*Shin Sai da kika haihu lafiya lau (Bayan kin fara yoyon) Ko kuwa an ciro jinjirin ne, ko anyi maki aiki ne?*	Normal delivery…………………………...1<br>Assisted vaginal delivery (forceps)………..2<br>C-section (delivery through the tummy)…..3
**304**	Have you ever sought treatment for this problem before arriving here for repair?<br><br>*Kin taba neman maganin wannan lalura kafin kizo ayi maki aiki?*	Yes……1<br>No……..2 → **skip to 401**

**305**	From whom did you seek treatment most recently (before arriving here for repair)?<br><br>*A wurin wa kika nemi magani kwanannan (kafin kizo wurin da za'a yi maki aikin)?*	Health professional (such as a doctor or nurse)……………………..……1<br>Community or village health worker..…………………………………2<br>Traditional healer, spiritual healer, native doctor, or other provider…..3<br>Other ...……………………………………………………………….4
**306**	Did any previous treatment involve surgery?<br><br>*A wurin neman magani an taba yi maki aiki?*	Yes……1 No…….2
**307**	How many times have you sought care for this condition (before coming here)?<br><br>*Sau nawa kika nemi kulawa saboda wannan yanayin (kafin kizo nan)?*	Write in:

SECTION 4: BARRIER CHECKLIST

After speaking to women in the area, we learned that there are a number of challenges that make it difficult to access repair care at facilities. Thinking back to your experience living with fistula, please tell us if you agree or disagree with the following statements. We will ask you if you strongly agree, agree, disagree, or strongly disagree.

SASHE NA HUHU: TAMBAYOYIN HANA NEMAN MAGANI

Bayan munyi Magana da mata acikin unguwa, mun fahimta cewa akwai kalubale da yawa da ke hana mata samun kuluwa idan anyi masu aiki a asibiti. Ki tuna saninki da yoyon fitsari, ki gaya mana kin yarda ko baki yarda ba akan tambayoyin da zamuyi maki yanzu.

	Question	Strongly Agree	Agree	Neither agree nor disagree	Disagree	Strongly disagree
**401**	I felt ashamed of having Obstetric Fistula (OF).<br><br>*Naji kunya dana kasance da lalurar yoyon fitsari.*					
**402**	I felt depressed and anxious.<br><br>*Na kaskanta kuma na damu.*					
**403**	I felt worthless.<br><br>*Naji kamar bani da muhimmanci.*					
**404**	I felt guilty because I had OF.<br><br>*Ina jin kamar bani da gaskiya don ina da ciwon yoyon fitsari.*					
**405**	I felt I am not as complete as a person because I had OF.<br><br>*Inajin ban cika mace ba don inada yoyon fistari.*					
**406**	Having OF made me feel unclean.<br><br>*Inajin kamar banda tsabta saboda lalura.*					
**407**	I depend on my husband or other male relatives for permission or money to seek care.<br><br>*Ina dogaro ne akan mijina ko yan uwana domin samun kudin magani ko kulawa.*					

**408**	I had family obligations that kept me from coming to the fistula centre.<br><br>*Ina da hidimar iyali ta dole dake hana ni zuwa cibiyar yoyon fitsari.*					
**409**	I prefer to use traditional medicine.<br><br>*Nafi bukatar son yin amfani da maganin gargajiya.*					
**410**	I fear and/or dislike hospitals and medical clinics.<br><br>*Inajin tsoron ko bani son asibiti da wurin bada magani.*					
**411**	I felt embarrassed because of my condition.<br><br>*Inajin na wulakanta saboda halin da nake ciki.*					
**412**	I felt isolated because of my OF condition.<br><br>*Inajin an maisheni saniyar ware saboda halin da nake ciki.*					
**413**	People who knew I had OF avoided me.<br><br>*Mutane sun guje ni saboda inada lalurar.*					
**414**	Women with OF are treated like outcasts.<br><br>*Mata masu irin wanan lalura an maida su kamar basu da daraja.*					
**415**	My husband/intimate partner treated me poorly initially.<br><br>*Maigida na da abokan zama sun maida ni maras galihu daga farko.*					
**416**	My husband/intimate partner treated me poorly later on.<br><br>*Mijina da abokan zama daga baya sun dauke ni akan maras galihu.*					
**417**	My husband/intimate partner abandoned me.<br><br>*Mijina da abokan zama sun guje ni.*					

**418**	I did not have someone to care for me and help me manage my condition at home.<br><br>*Bani da wanda zai taimakeni don inje cibiyar yoyon fitsari don samun magani da kulawa.*					
**419**	I did not have someone to support me in seeking and reaching care at the fistula centre.<br><br>*Bani da wanda zai taimake ni da cewa ciwon yoyon fitsari na bukatar zuwa asibiti don neman magani.*					
**420**	I was told by other people that my fistula would heal itself.<br><br>*Wasu mutane sun gayaman cewa ciwon zai warke don kansa.*					
**421**	I did not know that fistula is a medical condition that can be treated.<br><br>*Ban san cewa ciwon yoyon fitsari na bukatar zuwa asibiti ba don magancewa ba.*					
**422**	I believed that having OF was a curse.<br><br>*Nayi imani cewa ciwon yoyon fitsari baki ne.*					
**423**	I believed that my OF was caused by diabolic means.<br><br>*Nayi imani cewa ciwon yoyon fitsari sammu ne.*					
**424**	I did not know where to go for fistula repair.<br><br>*Basan wuri da zanje domin yimani aiki ba.*					
**425**	I did not have money to pay for medical care to treat my fistula.<br><br>*Bani da kudin da zan biya ma kaina magani.*					
**426**	I was unable to work because of the discomfort caused by my OF condition.<br><br>*Bani iya aiki saboda rashin jin dadi dangane da ciwon yoyon fitsari da nake dashi.*					

**427**	I was unable to work because of stigma associated with my OF condition.<br><br>*Bani iya aiki saboda kyamar da ake nuna mani ta ciwon yoyon fitsari.*					
**428**	Once others learnt of my condition, they did not allow me to work/earn money.<br><br>*In dai har wasu suka ji halin da nake ciki, to baza su barni inyi aiki ko in samu kudi ba.*					
**429**	The cost of transportation to repair sites and accommodation was too high.<br><br>*Kudin sufuri da wurin kwana zuwa wurin da ake aiki yayi yawa.*					
**430**	There are many transport options to get to the fistula centre.<br><br>*Hanyar zuwa asibiti tayi nisa.*					
**431**	The repair facility was too far.<br><br>*Asibitin da ake aikin tayi nisa.*					
**432**	The road conditions were bad.<br><br>*Hanyar zuwa asibiti bata da kyau.*					
**433**	I fear traveling to the health facility because of pain and discomfort during travel.<br><br>*Ina tsoron zuwa asibitin saboda inajin zafi da rashin jin dadi wajen tafiya.*					

**434**	I felt embarrassed about smell/leaking while traveling to the facility.<br><br>*Inajin kunya saboda wari da yoyon fitsari idan ina tafiya.*					
**435**	Repair facilities have enough doctors and nurses.<br><br>*Asibiti da ake aikin suna da isassun likitoci da masu jinya.*					
**436**	I worried that there are not many female health workers at the facility.<br><br>*Na damu saboda babu isassun mata masu aikin kiwon lafiya a asibitin.*					
**437**	Facilities do not have electricity, equipment, and/or supplies to provide health services.<br><br>*Babu wutar lantarki da sauran kayan aiki don bayar da kiwon lafiya.*					
**438**	I was afraid of harsh treatment by providers at the fistula centre.<br><br>*Ina tsoron wulakancin ma'aikatan kiwon lafiya asibitin.*					
**439**	I did not want to stay for a long time at the fistula centre to receive care.<br><br>*Bani son in tsaya lokaci mai tsawo don samun kula a asibitin.*					
**440**	I was told by health providers (in the past) that my fistula will heal itself.<br><br>*Masu kiwon lafiya sun goya man tun farko cewa ciwo na zai warke don kansa.*					
**441**	I was told by health providers (in the past) that my fistula cannot be treated.<br><br>*Masu kiwon lafiya sun gaya mani tun farko cewa ciwo na ba'a iya magance shi.*					

**442**	When I went to other facilities for a different health condition, doctors and nurses did not treat me properly.<br><br>*Naje wata asibiti da matsala ta daban amma ban samu kulawa sosai ba.*					
**443**	Repair /treatments I had in the past did not work.<br><br>*Aikin da akayi man na farko bai yiba.*<br><br>[ask only if woman has had repair in the past]					

SECTION 5: OPEN-ENDED QUESTIONS

Now we would like to hear **in your own words** about the barriers you faced and your experience of repair. Is it alright with you if I record this conversation so that I don't miss anything?

SASHE NA BIYAR: BUDADDIYAR TAMBAYA(KEKE – DA KEKE)

Yanzu inason inji daga bakin ki akan tarnakin da ake samu wajen yin aikin yoyon fitsari. Kin yarda in dauki mur yarki don kada in mance wani abu?

501. a. Describe any other barriers you faced in getting to the fistula center for repair? [Probe: cultural, religion, gender, social norms, family, poverty, stigma]

Kiyi man bayanin abinda yasa ya kawo maki tarnaki ko hannaki zuwa cibiyar da ake aikin. (Tabbatas: al adu, addini, mace ko namiji, Yanayin rayuwa, iyali, Talauci, tsana (Kyama)

b. Any barriers you faced after reaching the facility? [Probe: nurses, doctors, other health officers, anything else]

Akwai wata Katanga da kika fuskanta lokacin da kikaje asibiti? (Tabbatas masu aikin jinya likitoci, masu aikin kiwon lafiya, ko wani abu).

502. How did you get information about the service center? [Probe: from who, where]

Ta yaya kika samu bayanin cibiyar dake wanna aiki?

503. Who are the people who helped you to get to the facility? [Probe: family member, friend, community-based agent, primary health center worker, other]

Wane mutane suka taimakeki zuwa wanna asibitin (Tabbatas: Cikin iyali, abokai, yan unguwa, Ma'aikatan asibiti).

504. Did you receive a referral to this fistula center? If so, please describe referral process. [Probe]

Shin anturo ki ne zuwa wannan asibiti (Cibiyar) idan hakane, kiyi bayani yadda aka turo ki.

505. Describe how you came to the fistula center? [Probe, follow up on anything said early, confirm]

Kiyi mani bayanin yadda kika zo wanna cibiyar ta yoyon fitsari (Tabbatas :ta dalilin bayanin wani)

506. Who paid for your transportation to this fistula center? [Probe: Myself, Family member or accompanying person, Fistula center staff, other]

Wa yabiya maki kudin sufurin da kika zo wannan cibiyar ta yoyon fitsari? (Tabbatas: Dan uwa, abokin tafiya, Ma'aikacin cibiyar, Ko wa?)

507. How is your relationship with your husband/intimate partner? [Probe: interactions, positive experience, negative experience, how it makes you feel]

Yaya dangantakar ki take da Mijinki/ko abokiyar zama? (Tabbatas: Ana hudda, inganci, Maras inganci ya kikeji)

508. Now that your fistula has been repaired, do you think that your relationship with your husband/intimate partner will change? How? [Probe]

Yanzu tunda anyi maki aiki yayi kyau, kinajin dangantakarki da mijin ki zata chanja? Ta yaya?

509. How is your relationship with your family and friends? [Probe: interactions, positive experience, negative experience, how it makes you feel]

    Ya dangantakar ki take da iyali da sauran abokai?

510. Do you think your relationship with family and friends will change now that you are repaired? How? [Probe: social, relationships, interactions]

    Kinajin dangantakar ki da iyali da sauran abokai zata gyaru tunda anyi maki aiki? Ta yaya?

511. Do you know any other person having similar symptoms as you?

    Kinsan wata mace mai irin alamomin kamar naki?

512. What do you feel would be a good way for a person with your symptoms to get information about services for treatment? [Probe: from who, how, mode of communication, outreach]

    Wace hanyace mai kyau da kike ganin mace mai alamomi irin naki zata samu bayani akan bada magani ta wannan ciwon? (Tabbatar: Daga wa, ta yaya, ta wane bayani ko sadarwa, ko sai an tsallaka kauye)

Do you have any other comments for us at this time?

Kina da wani bayani da kike son kiyi yanzu?

Thank you for answering these questions for us.

Nagode da kika amsa mani wadannan tambayoyi.

Women's Interview

Fistula Care *Plus* – Baseline – Ebonyi

Women's post repair interview/barrier checklist (administered during stay at fistula centre)

QUESTIONNAIRE IDENTIFICATION INFORMATION

Questionnaire Number*:* [ ][ ][ ][ ][ ][ ][ ]

INTERVIEWER: CODE [____|____]   NAME___________________________
DATE OF INTERVIEW: __/____ /_____
TIME INTERVIEW STARTED: HOUR/MINUTES: |___|___| (USE 24 HOURS CLOCK)
SUPERVISOR'S REVIEW: (NAME) ___________________
Signature after editing ________________ DATE __________________
COUNTRY: Nigeria (01)   Uganda (02)   CODE |___|___|
STATE: Ebonyi (01)   Katsina (02)   **CODE |___|___|**

Local Governmental Area (LGA): ___________________________________

Venue of Interview_____________________________ (Write name of facility or Division & Location)

Informed Consent Form signed Yes - 1 No - 2
Language used to conduct interview: 1-English, 2- Igbo, 3– Hausa, 4—Combined 5—Other (specify)
____

Interview result: Completed – 1   Postponed – 2   Refused – 3 Incomplete---4

If incomplete (reason____)

Under formatting review

Women's Interview

	**SECTION 1: SOCIO DEMOGRAPHIC CHARACTERISTICS**<br>First I would like to ask some questions about you and your household.<br>*Na mbu, achorom iju gi ajuju gbasara gi na ezi na ulo gi*	
**101a**	From the time you were born till you were 12 years, did you live in town or in the rural areas?<br><br>*Malite mgbe amuru gi rute mgbe idi afo iri na-abua, ibi na nnukwu obodo ka obu n'ime ime obodo?*<br><br>[READ OUT OPTIONS]	District town………..1<br>Other city or town…..2<br>Rural areas ………….3<br>Outside Ebonyi ……...4
**101b**	Where are you living now?<br><br>*Kedu ebe ibi ugbua?*<br><br>[READ OUT OPTIONS]	District town………..1<br>Other city or town…..2<br>Rural areas ………….3 Outside Ebonyi……...4
**102**	How long have you been living in that area (before coming to fistula center)?<br><br>*Ibiela ebe ahu ruo afo ole?*<br><br>[WRITE IN YEARS OR CIRCLE]	Years (specify_____________)…1<br>Always……………………………2<br>Visitor ……………………………3
**103**	In what MONTH and YEAR were you born?<br><br>*Kedu mgbe a'muru gi? Kedu Onwa na afor amuru gi?*	**MONTH** [ ]<br>know month ….. 98<br>**YEAR** [ ]<br>know year……… 8
**104**	How old were you at your last birthday?<br><br>*Idi afo ole mgbe imere emume agbam afo gi nke ikpe azu?*<br><br>[WRITE IN. PLEASE RECONCILE IF THERE IS A DIFFERENCE BETWEEN q103 AND q104]	**Years** [ ]<br><br>Don't know……………………..98

Under formatting review

**105**	Have you ever attended school?<br>*Igatula ulo akwukwo?*	Yes…………1<br>No…………..2---------**Skip to Q109**
**106**	What is the highest level of school you attended: Primary, secondary, or more than secondary?<br>*Kedu ebe igudebere n'ulo akwukwo? Prima? Ogo sekondiri?*<br>*Ka igugoo karia nke secondary?*	Primary .... 1<br>Secondary .... 2<br>More than secondary 3

**107**	What is the highest you completed at that level?<br>*Olee ebe ikwusiri n'ulo akwukwo?*<br>[WRITE IN]	Standard/form/class ___________<br>Don't know ……………………….99
**109**	What is your religion?<br>*Kedu okpukpere chi nke ikwenyere ?*	Catholic………………………………1<br>Other Christian……………………….2<br>Islam………………………………….3<br>Traditionalist…………………………4<br>No religion …………………………..5<br>Other (Specify)_________________....6
**110**	What is your ethnic group?<br>*Olee agburu gi: ibu onye Awusa, Yoruba ka ibu onye Igbo?*	Hausa…………………………..1<br>Igbo……………………………....2<br>Yoruba…………………….…….3<br>Fulani…………………….…..…4<br>Ibibio…………………….…..…5<br>Ijaw…………………..……...6<br>Kanuri……………….................7<br>Tiv……………………………....8<br>Other (specify______________)….9
**111**	Aside from your normal housework are you currently working?<br>*Ewezugakwa oru ina aru n'ulo gi, onwere oru ozo ina-aru?*	Yes…….1 **Skip to Q 113**<br>No……..2 **Skip to Q 112**
**112**	Aside from your normal housework, have you worked for income/pay in the last 12 months?<br>*Nime onwa iri n'abuo gara aga, onwegoo oru ozo iruru maka ego?*	Yes………1 **Skip to Q 113**<br>No……….2 **Skip to Q 117**

**113**	What is your occupation, that is, the kind of work you normally do?<br><br>*Gini ka ina-aru?*<br><br>[WRITE IN]	Write in:
**114**	What is your monthly income?<br><br>*Obu ego ole ka ina akpata n'onwa?*<br><br>[IF DON'T KNOW, READ OUT]	Below NGN 5000…………….1<br>NGN 5000-10,000……………2<br>NGN 10,000-15,000 …………3<br>NGN 15,000-20,000…………4<br>NGN 20,000-25,000…………5<br>NGN 25,000-30,000…………6<br>NGN 30,000-35,000…………7<br>NGN 35,000-40,000…………8<br>NGN 40,000 and above………9<br>Don't know……………………98

**115**	Who mainly decides how the money you yourself earn is spent?<br><br>*Obu onye na'ekpebi etu isi emefu ego ina apkata*<br>[READ OUT]	Self……………………………….1<br>Husband/partner…………………..2<br>Self and husband/partner jointly….3<br>Someone else……………………..4<br>Self and someone else jointly ……5<br>N/A……………………………….99
**116**	On average how much of your household's expenditure do your earnings pay for: almost none, less than half about half, more than half or all?<br><br>*Kedu ebe ugwo onwa gi n'a ejedebe igborogi mkpa ezi na ulo gi?*<br><br>[READ OUT]	Almost none…………………1<br>Less than half………………..2<br>About half……………………3<br>More than half………………..4<br>All……………………………5<br>None her income is saved…….6
**117**	What is your marital status?<br><br>*Ichibago ukwu n'ulo; ilugoo di m'obu nwunye?*	Single (Never Married)……1 **skip to 201**<br>Cohabiting…………………2 **Skip to Q 118**<br>Married……………………3 **Skip to Q 118**<br>Separated…………………..4 **skip to 119**<br>Divorced…………………...5 **skip to 119**<br>Widowed…………………..6 **skip to 119**

**118**	Is your husband/partner living with you now or is he staying elsewhere?<br>*Gi na di gi m'obu onye nke gi ebi otu ebe ugbua ka onwere ebe ozo obi?*	Living with her ……1 Staying elsewhere….2
**119**	Does/did your husband/partner have any other wives beside yourself?<br>*Nna gi ukwu, onwere ndi nwunye ozo wezuga gi onwe gi?*	Yes………….1<br>No……………2 **--------Skip to 121**<br>Don't know….98
**120**	Are/were you the first wife or second or.....?<br>*Ibu isi nwanyi nna gi ukwu, ka ibu onye nke abua?*	Only wife…………1<br>First wife …………2<br>Second wife……….3<br>Third wife…………4<br>Fourth wife ……….5<br>Don't know………..98
**121**	When you first got married or lived with a man was it your choice, or did your family arrange it?<br>*Mgbe mbu iluru di, mobu mgbe mbu gi na nwoke biwara n'ulo, obu site n'uche aka gi, ka obu ndi ezinulo unu kwanyere gi nime ya?*	Own choice……………1<br>Arranged by family ……2
**122**	When you first got married or lived with a man: was the man younger, about the same age or older than you?<br>*Ngbe iluru di m'obu mgbe gi na nwoke malitere biwe, gi na nwoke ahu gina ya biri dika di na nwunye, obu onye toro ibe ya? Ka unu abu ogbo?*<br>[PROBE: IF OLDER, HOW MUCH OLDER THAN YOU, LESS THAN 10 YEARS OR MORE THAN 10 YEARS OLDER?]	Younger……………………………1<br>About the same age…………………2<br>Older – less than 10 years older…….3 Older - more than 10 years older ….4<br>Don't know ………………………..98

**123**	How old were <u>you</u> when you first got married or started living with a man?<br><br>*Idi afo ole wee luo di, ma obu mgbe gi na nwoke malitere biwe?*	Age in years [ ][ ]<br><br>Can't remember……………..98
**124**	Did your husband ever attend school?<br><br>*Di gi ahu oguru akwukwo?*	Yes……………..1<br>No………………2---------**skip to 201**<br>Don't know…….98----------**skip to 201**
**125**	What was the education level of your husband?<br><br>*Ebee ka di gi ahu gudebere n'akwukwo?*<br><br>[READ OUT]	Primary……………………....1<br>Secondary……………………2<br>More than secondary………..3<br>Don't know…..……………98 →**skip to 127**
**126**	What is the highest your husband completed at that level?<br><br>*Asambo gini ka di gi ji ugbua?*<br><br>[WRITE IN]	Standard/form/class ________<br><br>Don't know………………..98
**127**	What is the current occupation of your husband, that is, what kind of work does he mainly do?<br><br>*Gini ka di gi n'aru ugbua?*<br><br>[WRITE IN]	Write in:

SECTION 2: REPRODUCTIVE AND MATERNAL HISTORY<br>Now I would like to ask you about pregnancy, childbirth, and also some questions about sexual activity and reproductive health in order to better understand some family life issues.<br><br>*Ugbua oga amasim iju gi maka afor ime, imu nwa na mmeko nwoke na nwanyi, ahu ike otutu na omumu ka anyi ghota ihe gbasara ezi na ulo.*		
**201**	How old were you when you first had sexual intercourse?<br><br>*Mgbe izizi gina nwoke nwere mmeko, idi afo ole?*<br><br>[PROBE: WHAT WAS TYPICAL AGE FOR GIRLS TO GET MARRIED]	10-14 years……………………….1<br>15-19 years……………………….2<br>20-24 years……………………….3<br>25 years and above……………….4
**202**	How old were you when you became pregnant the first time?<br><br>*Idi afo ole mgbe ituru ime mbu gi?*	10-14 years……………………….1<br>15-19 years……………………….2<br>20-24 years……………………….3<br>25 years and above……………….4
**203**	At the first time you became pregnant, did you <u>want</u> to be pregnant then, did you want to <u>wait until later</u> or did you <u>not want</u> to have the baby at all?<br><br>*N'oge mbu ahu idi ime, ichoro ituru ime mgbe ahu, ka ichoro icheretu emechaa na mgbe di nihu, ka obu na ichoghi imu nwa ahu?*	Wanted it then…………………1<br>Wait till later…………………...2<br>Did not want …………………...3
**204**	How many pregnancies have you had? Including those that miscarried?<br><br>*Ugboro ole ka itugoro ime, ma ndi nke puru apu?*<br><br>[WRITE IN NUMBER]	Write in:

**205**	How many living children do you have now? (biological children only)<br><br>*Umu gi ole no ndu taa?*	Write in:
**206a**	During your last(full-term) pregnancy, did you attend antenatal care?<br><br>*Afor ime ikpe azu ibuzuru muo, ijere ulo ogwu maka ante natal?*	Yes…………1<br>No………….2**----------skip to 207a**
**206b**	How many times did you attend antenatal care?<br><br>*Ugboro ole ka igara antenatal ?*	Once ………………..1<br>Twice ………………2<br>Three times…………3<br>Four times …………..4<br>Five or more………....5
**207a**	Where did you deliver?<br><br>*Kedu ebe imuru nwa gi?*<br><br>[READ OUT]	At hospital/facility/PHC……….1<br>At home………………………..2<br>At home with TBA……………..3
**207b**	Did you suffer any complications?<br><br>*Inwere nsogbu obula mgbe ahu?*<br><br>[READ OUT, CIRCLE ALL THAT APPLY]	Bleeding…………………………1<br>Prolongedlabour………………...2<br>Fitting……………………………3<br>Sepsis …………………………...4<br>Other ……………………………5<br>(Specify_________________)<br>None ……………………………6
**207c**	What was your delivery outcome?<br><br>*Gini bu onodu nwa ahu imuru?*	Live baby……………………………….1<br>Live baby but died few hours days later….2<br>Stillbirth………………………………..3

	SECTION 3: FISTULA<br>Now I'm going to ask you a few questions about the development of your fistula.<br><br>*Ugbua, anam aga iju gi ufodu ajuju gbasara etu oria ngbapu akpa mamiri gi si bido.*	
**301**	How long had you been living with fistula?<br><br>*Omegoo afo ole iriawara oria mgbapu akpa mamiri?*<br><br>[WRITE IN]	Write in:
**302**	When did this problem (LEAKAGE OF URINE AND/OR FECES) start?<br><br>*Kedu mgbe oria malitere riawa gi?*<br><br>[LET WOMAN RESPOND; IF SHE IS SILENT, READ OUT]	After you delivered a live or stillborn baby………………………..1<br>After abdominal or pelvic surgery while you were not pregnant….2 → **skip to 304**<br>After a sexual assault, attack, or other injury……………………...3 →**skip to 304**<br>None of the above …………………………………………………4→**skip to 304**<br>Don't know……………………………………………………….99 →**skip to 304**
**303**	Did this delivery (after which leaking started) happen normally, did they pull the baby out, or did they cut you/do an operation?<br><br>*Nwa ahu imuchara malite inwe nsogbu mgbapu akpa mamiri, imuru ya n'udo, ka ha eji ike doputa nwa ahu, ka eji aguba yi waa gi ahu nwanyi gi, ka ha akpara gi mma n'afo?*	Normal delivery…………………………...1<br>Assisted vaginal delivery (forceps)………..2<br>C-section (delivery through the tummy)…..3
**304**	Have you ever sought treatment for this problem before arriving here for repair?<br><br>*Onwego ebe ozo igara nara ogwu maka nsogbu gia, tupu ibia ebe a?*	Yes……1<br>No……..2 → **skip to 401**

**305**	From whom did you seek treatment most recently (before arriving here for repair)?<br><br>*Kedu udi ndi igara na nke ha ka ha nyere gi aka n'oria ahu tupu ibia ebea ka edozie gi onodua?*	Health professional (such as a doctor or nurse)…………………..……1<br>Community or village health worker..…………………………………2<br>Traditional healer, spiritual healer, native doctor, or other provider…..3<br>Other ...………………………………………………………………4
**306**	Did any previous treatment involve surgery?<br><br>*Onwere ka ha sigoo kpaa gi mma n'ahu, n'udi na ha nagwo gi oria?*	Yes……1<br>No…….2
**307**	How many times have you sought care for this condition (before coming here)?<br><br>*Ugboro ole ka igagoro ka agwoo gi oria, tupu i bia ebea?*	Write in:

SECTION 4: BARRIER CHECKLIST					
After speaking to women in the area, we learned that there are a number of challenges that make it difficult to access repair care at facilities. Thinking back to your experience living with fistula, please tell us if you agree or disagree with the following statements. We will ask you if you strongly agree, agree, disagree, or strongly disagree.<br><br>*Dika anyi na umu nwanyi no ogige ebea kpachara, anyi choputara na enwere ihe ole na ole na echere ha aka. Mgba mere oji esiri ha ike ibia nata ogwugwo na ulo ogwu. Iche laa azu na ihe megorogi maka inwe mgbapu akpa mamiri, biko gwa anyi ma ikwenyere ma obu ju okwu ndia. Anyi ya aju gi ma ikwenyesiri ike, ikwe nyere, ijuru ma obu ijusi ike.*					
	**Question**	**Strongly Agree**	**Agree**	**Disagree**	**Strongly disagree**
**401**	I felt ashamed of having Obstetric Fistula (OF).<br>*Ihere mere m, maka oria mgbapu akpa mamiri ahu.*				
**402**	I felt depressed and anxious.<br>*Obi na amapuwam mgbe obula, anam adawa mba no'bim oge ahu.*				
**403**	I felt worthless.<br>*Odim ka obu na enwighi uru mbara.*				
**404**	I felt guilty because I had OF.<br>*Anam echewa na ikpe maaram maka oria mgbapu akpa mamiri m ahu.*				
**405**	I felt I am not as complete as a person because I had OF. *Ona-adiwam ka aga asi na ezughim oke mmadu maka yabu mgbapu akpa mamiri m.*				
**406**	Having OF made me feel unclean.<br>*Ona-adi m ka obu na 'adighim cha.*				
**407**	I depend on my husband or other male relatives for permission or money to seek care.<br>*Adabeerem n'aka dim m'obu umunnem ndi nwoke ozo maka ikike ya na ego m ji elebara onwem anya.*				

**408**	I had family obligations that kept me from coming to the fistula centre.<br>*Onwegasiri mkpa ezinulo dikariri onodum ahu mkpa mere mji agaghim ulo ogwu.*				
**409**	I prefer to use traditional medicine.<br>*O kaara m mma inarawa ogwu igbo.*				
**410**	I fear and/or dislike hospitals and medical clinics.<br>*Ujo ulo ogwu n'atuwa m, obim anabataghi ulo ogwu bekee*<br>.				
**411**	I felt embarrassed because of my condition.<br>*Onodu m ahu na eme m ihere.*				
**412**	I felt isolated because of my OF condition.<br>*Ona-adim ka ana asom aso maka mgbapu akpa mamirim ahu.*				
**413**	People who knew I had OF avoided me.<br>*Ndi matara na enwerem mgbapu akpa mamiri anaghi eru m nso.*				
**414**	Women with OF are treated like outcasts.<br>*Umunwanyi ndi nwere mgbapu akpa mamiri; ka ana ahutawa dika ndi ajuru aju n'obodo.*				
**415**	My husband/intimate partner treated me poorly initially.<br>*Na mbido, dim m'obu onye nkem anaghi elebaram anya etu okwesiri.*				
**416**	My husband/intimate partner treated me poorly later on.<br>*Di m m'obu onye nkem anaghi elebaram anya etu okwesiri ugbua.*				
**417**	My husband/intimate partner abandoned me.<br>*Di m, m'obu onye nkesm gbahapurum.*				
**418**	I did not have someone to care for me and help me manage my condition at home.<br>*Na ulo, enweghi onye obula na enyere m aka ma obu letem n'onodu m ahu.*				

**419**	I did not have someone to support me in seeking and reaching care at the fistula centre.<br>*Onweghi enyemaka maobu nkwado obula m'nwere, maka ichota ebe aga elebara onodum ahu anya.*				
**420**	I was told by other people that my fistula would heal itself.<br>*Ndi ozo gwaram na oria mgbapu akpa mamirim ga ala na onwe ya.*				
**421**	I did not know that fistula is a medical condition that can be treated.<br>*Amaghim na mgbapu akpa mamiri bu oria enwere ike igwo n'ulo ogwu.*				
**422**	I believed that having OF was a curse.<br>*Ana m echewa na onye n'aria oria mgbapu akpa ma miri, bu onye aburu onu.*				
**423**	I believed that my OF was caused by diabolic means.<br>*Odim ka obu ihe aka mere.*				
**424**	I did not know where to go for fistula repair.<br>*Onweghi ebe m mara m ga-aga ka 'edozie akpa ma miri m ahu gbapuru.*				
**425**	I did not have money to pay for medical care to treat my fistula.<br>*Enweghi m ego mji akwu ugwo maka imebe m ogwu mobu gwoo m oria ahu.*				
**426**	I was unable to work because of the discomfort caused by my OF condition.<br>*Enweghim ike iru oru maka mmekpa ahu oria ahu na ewetara m.*				
**427**	I was unable to work because of stigma associated with my OF condition.<br>*Enweghi m ike ruo oru maka mkpachapu ana emem n'ebe m na aru oru.*				

**428**	Once others learnt of my condition, they did not allow me to work/earn money.<br>*Ozigbo ndi mmadu ozo choputara onodu m ahu, ha enweghizi m ohere iru oru m'obu ikpata ego.*				
**429**	The cost of transportation to repair sites and accommodation was too high.<br>*Ugwo iga ebe aga edozim akpa mamiri yana ugwo ulo ebe m ga ano ma m'gaa di oke onu.*				
**430**	There are many transport options to get to the fistula centre.<br>*Enwere otutu uzo ndi ozo a ga esi gaa ulo ogwu ahu.*				
**431**	The repair facility was too far.<br>*Ebe ana agwo oria ahu di anya.*				
**432**	The road conditions were bad.<br>*Uzo esi aga ebe ahu adighi mma.*				
**433**	I fear traveling to the health facility because of pain and discomfort during travel.<br>*Ujo n'atu m iga ebe ana agwo oria ahu maka ihe mgbu na mmekpa ahu m' na-agabiga.*				
**434**	I felt embarrassed about smell/leaking while traveling to the facility.<br>*Oke isi m na-esi ya na mamiri sim n'ahu 'edeputa na emem ihere.*				
**435**	Repair facilities have enough doctors and nurses.<br>*Ebe ana agwo yabu oria nwere ndi dibia oyibo na ndi oru nurses zuru oke.*				
**436**	I worried that there are not many female health workers at the facility.<br>*Ocherem uche na enweghi otutu ndi nwanyi oru ahuike naaru oru ebe ahu.*				

**437**	Facilities do not have electricity, equipment, and/or supplies to provide health services.<br>*Ulo ogwu ahu enweghi oku latrik, ha enweghikwa mgwomgwo zuru oke eji agwo oria ahu.*				
**438**	I was afraid of harsh treatment by providers at the fistula centre.<br>*Ujo n'turum maka omume obi ojoo ndi oru ahu n'emeso ndi oria.*				
**439**	I did not want to stay for a long time at the fistula centre to receive care.<br>*Achoghim inote aka tupu elebaram anya.*				
**440**	I was told by health providers (in the past) that my fistula will heal itself.<br><br>*N'oge gara aga, ndi oru ahuike gwara m na akpa mamiri m ahu gbapuru, ga emechaa were aka ya mechie.*				
**441**	I was told by health providers (in the past) that my fistula cannot be treated.<br><br>*N'oge mbu ndi ahuike gwara m na anaghi agwo oria ahu agwo.*				
**442**	When I went to other facilities for a different health condition, doctors and nurses did not treat me properly.<br>*Mgbe m gara ulo ogwu ndi ozo maka oria ozo ji m aka, ndi dibia bekee na ndi nurse elezighi m anya nke oma.*				
**443**	Repair /treatments I had in the past did not work.<br>*Ndozi edoziburu akpa mamiri m 'ahu mgbe mbu enweghi isi.*<br>[ask only if woman has had repair in the past]				

Under formatting review

SECTION 5: OPEN-ENDED QUESTIONS

Now we would like to hear **in your own words** about the barriers you faced and your experience of repair. Is it alright with you if I record this conversation so that I don't miss anything?

Ugbu anyi ga acho inuta uche na nghota nke gi banyere ogbata uhue inwere ma obu ihuru mgbe ana ugwo gi oria.Oga adi gi mma mam were igwea gete olu gi, kam ghara ichefu ihe obula?

501. a. Describe any other barriers you faced in getting to the fistula center for repair? [Probe: cultural, religion, gender, social norms, family, poverty, stigma]

 Kowaara anyi, ogbata uhie ndi ahu izutere , mgbe i na aga n'ogige ebe ahu ana edozi akpa mamiri?

 b. Any barriers you faced after reaching the facility? [Probe: nurses, doctors, other health officers, anything else]

 Onwere ogbata uhue izutere mgbe irutere ebe ahu?

502. How did you get information about the service center? [Probe: from who, where]

 Kedu ka isiri nu ihe banyere ogige ebe ahu?

503. Who are the people who helped you to get to the facility? [Probe: family member, friend, community-based agent, primary health center worker, other]

 Onye bu onye ahu nyeere gi aka igaru n'ogige ahu?

504. Did you receive a referral to this fistula center? If so, please describe referral process. [Probe]

 Inatara ozi ntuzi aka maka ogige ahu? Oburu na aziza gi bu eeh; gwakene anyi ka isiri nata ntuzi aka ahu?

505. Describe how you came to the fistula center? [Probe, follow up on anything said early, confirm]

Kowaram ka isiri rute nogige ahu?

506. Who paid for your transportation to this fistula center? [Probe: Myself, Family member or accompanying person, Fistula center staff, other]

Onye kwuru gi ugwo ugbo ala butere gi nogige ahu?

507. How is your relationship with your husband/intimate partner? [Probe: interactions, positive experience, negative experience, how it makes you feel]

Kedu ka mmekorita gi na di gi ma obu onye nke gi di?

508. Now that your fistula has been repaired, do you think that your relationship with your husband/intimate partner will change? How? [Probe]

Dika agwogoro gi mgbapu akpa mamiri gi ahu, odi gi ka mmeko di n;etiti gi na di gi ma obu onye nke gi oga agbanwe? Kedu etu obu?

509. How is your relationship with your family and friends? [Probe: interactions, positive experience, negative experience, how it makes you feel]

Kedu ka mmekorita gi na ndi ezi na ulo gi ma obu ndi enyi gi di?

510. Do you think your relationship with family and friends will change now that you are repaired? How? [Probe: social, relationships, interactions]

Odi gi ka mmeko din a etiti gi na ndi ezinulo gi na ndi enyi gi oga-agbanwe? Kedu n'udi obu?

511. Do you know any other person having similar symptoms as you?

Onwere onye ozo imara, nwere udi nsogbu gi a?

512. What do you feel would be a good way for a person with your symptoms to get information about services for treatment? [Probe: from who, how, mode of communication, outreach]

Kedu etu odi gi k'oga aka mma isi mee ka ndi mmadu nuta banyere ebe ana oria

Do you have any other comments for us at this time?

Onwere ihe ndi ozo inwere igwa anyi ugbua?

Thank you for answering these questions for us.

Women's Interview

Fistula Care *Plus* - Baseline – Uganda

Women's post repair interview/barrier checklist (administered during stay at fistula centre)

QUESTIONNAIRE IDENTIFICATION INFORMATION

Questionnaire Number*:* | | | | | | | [Facility code_Interviwer Code_Interview Number]

INTERVIEWER: CODE [____|____] NAME__________________________
DATE OF INTERVIEW: __/____ /_____
TIME INTERVIEW STARTED: HOUR/MINUTES: |___|___| (USE 24 HOURS CLOCK)
SUPERVISOR'S REVIEW: (NAME) ___________________
Signature after editing ________________ DATE __________________
COUNTRY: Nigeria (01) Uganda (02) CODE |___|___|
REGION: Central (01) Western (02) **CODE |___|___|**

District: ___________________________________

Venue of Interview____________________________ (Write name of facility or Division & Location) **Client status**: Post repair (01) Pre-repair/waiting (02)

If pre-repair/waiting, specify diagnosis: ________________________

Informed Consent Form signed Yes - 1 No - 2
Language used to conduct interview: 1-English, 2- Luganda, 3-Combined, 5-Other (specify)

____

Interview result: Completed – 1 Postponed – 2 Refused – 3 Incomplete---4

If incomplete (reason____)

	SECTION 1: SOCIO DEMOGRAPHIC CHARACTERISTICS<br>First I would like to ask some questions about you and your household.<br>*Okusookera ddala, njagala kukubuuzayo ebibuuzo ebikukwatako n'amakaago*	
**101a**	From the time you were born till you were 12 years, did you live in town or in the rural areas?<br><br>*Ebiseerabyo ebisinga bwewali nga tonnaweza myaka kumi n'ebiri (12) wabeeranga mu kibuga oba mu kyalo?*<br><br>[READ OUT OPTIONS]	District town………………………..1<br>Other city or town…………………..2<br>Rural areas ………………………....3<br>Outside Central-1 Sub Region ……...4
**101b**	Where are you living now?<br>Obeera wa ekiseeera kino?<br><br>[READ OUT OPTIONS]	Masaka…….…………………...........1<br>Kalungu………………………...........2<br>Other district (Specify)____________....3<br>Outside Central-1 Sub Region…………4
**102**	How long have you been living in that area (before coming to fistula center)?<br><br>*Mu kitundu kino wakabeerawo kumala bbanga ki?*<br><br>[WRITE IN YEARS OR CIRCLE]	Years (specify_____________)…1<br>Always……………………………2<br>Visitor ……………………………3
**103**	In what MONTH and YEAR were you born?<br><br>*Mwezi ki era Mwaka ki gwe wazaalibwamu?*	**MONTH** [ ]<br><br>know month …..<br><br>**YEAR** [ ]<br><br>know year………
**104**	How old were you at your last birthday?<br><br>*Waweza emyaka emeka ku mazaalibwa go agaakasemabayo?*<br><br>[WRITE IN. PLEASE RECONCILE IF THERE IS A DIFFERENCE BETWEEN q103 AND q104]	**Years** [ ]<br><br>Don't know……………………98
**105**	Have you ever attended school?<br>*Wali ogenze ko mu somero?*	Yes…………1<br>No…………..2---------**Skip to Q109**

Under formatting review

**106**	What is the highest level of school you attended: Primary, secondary, or more than secondary?<br>*Wasomako kyenkana wa: pulayimale, ssinya, oba okusukka ssiniya?*	Primary (specify:________) .... 1<br>Secondary (specify_______) .... 2<br>Tertiary/University (specify:______)… 3
**107**	What is your religion?<br>*Osoma ddiini ki?*	Catholic………………………………1<br>Protestant…….…………………………2<br>Muslim………………………………..3<br>Pentecostal……………………………4<br>SDA…………………………………..5<br>No religion …………………………...6<br>Other (Specify)_______________.....7
**108**	What is your ethnic group?<br>*Oli wa ggwanga/kabira ki?*	Baganda……………………………..1<br>Banyoro…………………………......2<br>Banyakole……………………..…….3<br>Basoga……………………..……..4<br>Bakiga……………………..……...5<br>Iteso……………………..……..........6<br>Acholi……………….........................7<br>Langi……………………………......8<br>Other (specify_____________)…….9
**109**	Aside from your normal housework are you currently working?<br>*Ng' ojjeeko emirimu gy'awaka, olina omulimu gw'okola?*	Yes…….1 **Skip to 111**<br>No……..2 **Skip to 110**
**110**	Aside from your normal housework, have you worked for income/pay in the last 12 months?<br>*Mu myezi kumi n'ebiri egyiyise, wakola emirimu egiyingiiza ensimbi?*	Yes………1 **Skip to 111**<br>No……….2 **Skip to 115**
**111**	What is your occupation, that is, the kind of work you normally do?<br>*Okola mulimu ki?*<br>[WRITE IN]	Write in:
**112**	What is your average monthly income?<br>*Oyingiza ssente mmeka omwezi?*<br>[Write in]	Don't know……………………98

**113**	Who mainly decides how the money you yourself earn is spent?<br>*Ani assinga okusalawo ku nsaasaanya y'ensimbi z'okola?*<br><br>[READ OUT]	Self……………………………….1<br>d/partner…………………..2<br>Self and husband/partner jointly….3<br>Someone else (specify:)……………..4<br>Self and someone else jointly (specify: _______)……5<br>N/A……………………………….99
**114**	On average how much of your household's expenditure do your earnings pay for: almost none, less than half about half, more than half or all?<br><br>*Okutwaliza awamu, ku nsimbi ezisaasaanyizibwa ku kukola ku byetaago by'awaka, oteekako mmeka: kyenkana tewali, wansi w'ekitundu, kyenkana kitundu, okusukka mu kitundu, zonna?*<br><br>[READ OUT]	Almost none…………………1<br>Less than half………………..2<br>About half……………………3<br>More than half………………..4<br>All……………………………5<br>None her income is saved…….6
**115**	What is your marital status?<br><br>*Oli mufumbo?*	Single (Never Married)……1 →**skip to 201**<br>Married/Cohabiting…………..2→**skip to 116**<br><br>Separated/Divorced…………..4 →**skip to 118**<br>Widowed…………………..6→**skip to 118**
**116**	Doesyour spouse have any other wives beside yourself?<br><br>*Balo alinayo abakyala abalala?*	Yes…………..1<br>No……………2 **--------Skip to 120**<br>Don't know….98 ----Skip to 120
**117**	Are you the first wife or second or.....?<br><br>*Oli mukyala wakumeka...?*	Only wife………..1 ----Skip to 120<br>First wife …………2 ----Skip to 120<br>Second wife……….3 ----Skip to 120<br>Third wife…………4 ----Skip to 120<br>Fourth wife ……….5 ----Skip to 120
**118**	Did your spouse have any other wives besides yourselves?<br>*Balo yalinayo abakyala abalala?*	Yes………..1<br>No…………2<br>Don't know…..98
**119**	Were you the first wife, second wife, or….?<br>*Owali mukyala wakumeka...?*	Only wife………..1<br>First wife …………2<br>Second wife……….3 Third wife…………4<br>Fourth wife ……….5

Under formatting review

**120**	When you first got married or lived with a man was it your choice?<br><br>*Lwewasookera ddala okufumbirwa oba okubeera n'omusajja, kyaalii kyeyagalire?*	Yes……………1<br>No……2
**121**	When you first got married or lived with a man: was the man younger, about the same age or older than you?<br><br>*Lwewasookera ddala okufumbirwa oba okubeera n'omusajja: omusajja yali muto kuggwe, mwali mwenkana oba yali mukulu kuggwe? (bwaba yali akusinga obukulu, yali akusinga kyenkana ki, wansi w'emyaka kkumi, oba okusukka mu myaka kkumi?)*	Younger……………………………1<br>About the same age…………………2<br>Older – less than 10 years older…….3<br>Older - more than 10 years older ….4<br>Don't know ………………………..98

	[PROBE: IF OLDER, HOW MUCH OLDER THAN YOU, LESS THAN 10 YEARS OR MORE THAN 10 YEARS OLDER?]	
**122**	How old were <u>you</u> when you first got married?<br><br>*Walina emyaka emeka lwewasookera ddala okufumbirwa?*	Age in years [ ][ ]<br><br>Can't remember……………..98
**123**	Did your husband ever attend school?<br><br>*Balo yasomako?*	Yes……………..1<br>No………………2---------**skip to 201**<br>Don't know…….98----------**skip to 201**
**124**	What is the education level of your husband?<br><br>*Balo yasomako kyenkana wa?*<br><br>[READ OUT]	Primary (specify: ________) ……........1<br>Secondary (specify: ______)………………………2<br>Tertiary/University (specify_________)…....3<br>……….4<br>Don't know…...……………98 →**skip to 127**
**125**	What is the current occupation of your husband, that is, what kind of work does he mainly do?<br><br>*Balo akola milimu ki, ntegeeza omulimu gw'asinga okukola?*<br><br>[WRITE IN, LIST ACCORDING TO PRIORITY]	Write in:

SECTION 2: REPRODUCTIVE AND MATERNAL HISTORY		
Now I would like to ask you about pregnancy, childbirth, and also some questions about sexual activity and reproductive health in order to better understand some family life issues.<br><br>*Kati njagala kukubuuza ebikwata ku mbuto, okuzaala, n'ebibuuzo ebikwata ku kwegatta mu by'omukwano, okusobola okutegeera ensonga z'obulamu bw'omu maka*		
**201**	How old were you when you first had sexual intercourse?<br>*Walina emyaka emeka lwewasookera ddala okwegatta mu by'omukwano?*	Age in years ☐☐
**202**	How old were you when you became pregnant the first time?<br><br>*Walina emyaka emeka lwewasookera ddala okufuna olubuto?*	Age in years ☐☐

**203**	At the first time you became pregnant, did you want to be pregnant then, did you want to wait until later or did you not want to have the baby at all?<br><br>*Lwewasookera ddala okufuna olubuto, wayagala okulufuna, wali oyagala okulindako okulufuna, oba wali toyagala mwana yadde?*	Wanted it then…………………1<br>Wait till later…………………...2<br>Did not want …………………...3
**204a**	How many pregnancies have you had?<br><br>*Wakafuna embuto emirundi emeka, nga n'ezavaamu ozitwaliddemu?*<br><br>[WRITE IN NUMBER]	Write in:
**204b**	How many pregnancies have you lost before 7 months?<br><br>Wakavamu embuto meka nga tezinaweza myezi musanvu?<br><br>[WRITE IN NUMBER]	Write in:

Under formatting review

**205**	How many children do you have? (biological children only)<br><br>*Olina abaana bameka?*<br><br>[WRITE IN NUMBER]	Write in:
**206a**	During your last pregnancy, did you attend antenatal care?<br><br>*Ku lubuto lwo olwakasembayo wagenda mu ddwaliro okunywa eddagala?*	Yes…………1<br>No…………2**----------skip to 207a**
**206b**	How many times did you attend antenatal care?<br><br>*Wagenda emirundi emeka mu ddwaliro okunywa eddgala?*<br>[WRITE IN NUMBER]	Write in:
**207a**	From where did you deliver?<br><br>*Wazaalirawa ?*<br><br>[READ OUT]	At hospital/facility/PHC…………………..1<br>At home…………………………………...2<br>With TBA ………...3<br>Other (specify: __________)……………………4
**207b**	Did you suffer any complications?<br><br>*Wafunamu obuzibu bwonna?*	Yes……………………..1<br>No………………………2 ----**skip to 207d**
**207c**	Which complications did you have?<br>*Wafunamu buzibu ki?*<br><br>[READ OUT, CIRCLE ALL THAT APPLY]	Bleeding…………………………1<br>Prolonged labour………………...2<br>Fitting……………………………3<br>Sepsis …………………………...4<br>Other ……………………………5<br>(Specify__________________)
**207d**	Was your baby ….?<br><br>*Omana wamuzaala mulamu…?*	Alive at birth……………………………….1<br>Alive, but died few hours later….2<br>Stillbirth………………………………..3

Under formatting review

SECTION 3: FISTULA Now I'm going to ask you a few questions about the development of your fistula. *Kati ngenda kukubuuzayo ebibuuzo ebikwata ku ngeri ekirwadde ky'okutonnya gyekyajjamu.*		
**301**	How long had you been living with fistula?<br><br>*Obulwadde bw'okutonnya omusulo oba obubi (Fistula) wakamalanabwo bbanga ki?*<br><br>[WRITE IN]	Write in:
**302**	When did this problem (LEAKAGE OF URINE AND/OR FECES) start?<br><br>*Ekizibu (KY"OKUTONNYA OMUSULO OBA OBUBI) kyatandika ddi?*<br><br>[LET WOMAN RESPOND; IF SHE IS SILENT, READ OUT]	After you delivered a live or stillborn baby……………………...1<br>After abdominal or pelvic surgery while you were pregnant………2<br>After abdominal or pelvic surgery while you were not pregnant….3 → **skip to 304**<br>After a sexual assault or attack……………………..4 →**skip to 304**<br>**After other injury/trauma……………………………………...5**<br>From birth…………………………………………………………..6<br>None of the above …………………………………………………7→**skip to 304**<br>Don't know…………………………………………………….99 →**skip to 304**
**303**	Did this delivery (after which leaking started) happen normally, did they pull the baby out, or did they cut you/do an operation?<br><br>*Okuzaala kuno (okwavaako okutonnya), wazaala bulungi, omwana baamusikayo oba baamukulongosaamu?*	Normal delivery…………………………...1<br>Assisted vaginal delivery (vacuum/forceps)………..2<br>C-section (delivery through the tummy)…..3<br>Abdominal surgery (for ruptured uterus)……4
**304**	Have you ever sought treatment for this problem before arriving here for repair?	Yes……1<br>No……..2 → **skip to 401**
	Wali onoonyezza obujjanjabi bw'ekizibu kino, nga tonnagya wano?	

**305**	From who did you seek treatment most recently (before arriving here for repair)?<br><br>*Obujjanjabi obwa sembayo ngo tonjjawano wafuna w'ani?*	Health professional (such as a doctor or nurse)……………………..……1<br>Community or village health worker..…………………………………2<br>Traditional healer, spiritual healer, native doctor, or other provider…..3<br>Other ...……………………………………………………………………4
**306**	Did any previous treatment involve surgery?<br><br>*Obujjanjabi bwonna bwewali ofunye, mwalimu okulongoosebwa?*	Yes……1 No…….2
**307**	How many times have you sought care for this condition (before coming here)?<br><br>*Mirundi emeka gyekanoonya obujjanjabi bw'ekizibu kino (nga tonnagya wano)?*	Write in:

SECTION 4: BARRIER CHECKLIST

After speaking to women in the area, we learned that there are a number of challenges that make it difficult to access repair care at facilities. Thinking back to your experience living with fistula, please tell us if you agree or disagree with the following statements. We will ask you if you strongly agree, agree, disagree, or strongly disagree.

Oluvannyuma lw'okwogerako n'abakyala b'omukitundu, tukizudde nti waliwo emiziziko egiwerako egizibuwuza okutuukirira ebifo awajjanjabirwa. Bw'olowooza ku by'oyiseemu nga olina ekirwadde ky'okutonnya, tusaba otubuulire oba okkiriziganya nabino wammanga oba nedda. Tujja kukubuuza oba okkiririza ddala, okkiriza, tokkiriza oba tokkizira nakamu.

	**Question**	**Strongly Agree**	**Agree**	**Neutral**	**Disagree**	**Strongly disagree**
**401**	I felt ashamed of having Obstetric Fistula (OF).<br>*Nawulira obuswavu olw'okuba n'obulwadde bw'okutonnya omusulo oba obubi(fistula).*					
**402**	I felt depressed and anxious.<br><br>*Nnawulira ennaku, n'okweralikirira*					
**403**	I felt worthless.<br>*Nawulira nga sirimu nsa n'obutaba namugaso.*					
**404**	I felt guilty because I had OF.<br>*Nafuna obutaba namirembe (okuwulira nga gwegusinga) olw'okuba nalina obulwadde bw'okutonnya (fistula)*					
**405**	I felt I am not as complete as a person because I had OF.<br>*Nawulira nga situkiridde nga abalala olw'okuba nnalina ekirwadde ky'okutonnya (Fistula*)					
**406**	Having OF made me feel unclean.<br>*Okubeera n'ekirwadde ky'okutonnya kyandeetera okuwulira nti siri muyonjo.*					
**407**	I depend on my husband or other male relatives for permission or money to seek care.<br>*Nnina okufuna olukusa oba ssente okuva ewa baze oba ab'oluganda abala abasajja okugenda okunnonya obujjanjabi*					

Under formatting review

**408**	I had family obligations that kept me from coming to the fistula centre.<br>*Nalina obuvunaanyizibwa bw'awaka obulala obwanemesa okuja ku ddwliro okfuna obujjanjabi.*					
**409**	I prefer to use traditional medicine.<br>*Nsinga kwagala kukozesa ddagala lya kinnansi*					
**410**	I fear and/or dislike hospitals and medical clinics.<br>*Ntya era/oba ssaagala malwaliro*					
**411**	I felt embarrassed because of my condition.<br>*Nawulira okuswala olw'embeera yange*					
**412**	I felt isolated because of my OF condition.<br>*Nawulira okusosolwa olw'embeera yange ey'ekirwaddw ky'okutonnya (Fistula)*					
**413**	People who knew I had OF avoided me.<br>*Abantu abaamanya nti nnina ekirwadde ky'okutonnya (Fistula) bannewala.*					
**414**	Women with OF are treated like outcasts.<br>*Abakyala abalina ekirwadde ky'okutonnya (fistula) batwalibwa nga ekyenyinyarwa.*					
**415**	My husband/intimate partner treated me poorly after I got fistula.<br>*Muksooka baze wange yampisa bubi nga nakafuna ekizibu ky'okutonya.*					
**416**	My husband/intimate partner treated me poorly later on when I had fistula.<br>*Olvanyuma baze wange yampisa bubi ekirwade ky'okutonya bwekyaandwako.*					
**417**	My husband/intimate partner abandoned me.<br>*Baze wange yandekawo*					
**418**	I did not have someone to care for me and help me manage my condition at home.<br>*Ssaalina annyamba kwanganga mbeera yange nga ndi waka*					

Under formatting review

**419**	I did not have someone to support me in seeking and reaching care at the fistula centre.<br>*Ssaalina annyamba mu kunoonya n'o kutuukirira bujjanjabi ewajjanjabibwa*					
**420**	I was told by other people that my fistula would heal itself.<br>*Abalala bangamba nti okutonya kuwona kwokka.*					
**421**	I did not know that fistula is a medical condition that can be treated.<br>*Nnali simanyi nti okutonnya (fistula) bulwadde obusobola okujjanjabwa.*					
**422**	I believed that having OF was a curse.<br>*Nnakkiriza nti okubeera n'obulwadde bw'okutonnya (fistula) kikolimo.*					
**423**	I believed that my OF was caused by diabolic means.<br><br>*Nalowoza nti okutonya kwajja nga eddogo.*					
**424**	I did not know where to go for fistula repair.<br>*Nnali simanyi wakugenda kujjanjabirwa kirwadde kya kutonnya (fistula)*					
**425**	I did not have money to pay for medical care to treat my fistula.<br>*Ssaalina ssente zakusasulira bujjanjabi bwa kirwadde kya kutonnya (fistula).*					
**426**	I was unable to work because of the discomfort caused by my OF condition.<br>*Nnali sisobola kukola olw'obutawulira bulungi obwaletebwa ekirwadde ky'okutonnya (fistula)*					
**427**	I was unable to work because of stigma associated with my OF condition.<br>*Sakolanga olwembeera yokusosolwa mubaantu olwekilwadde kino kyenalina.*					

**428**	Once others learnt of my condition, they did not allow me to work/earn money.<br>*Kasita abantu abalala baamanya embeera yange, tebanzikiriza kukola/ kwekolera nsimbi.*					
**429**	The cost of transportation to repair sites and accommodation was too high.<br>*Ebisale by'entambula okugenda awajjanjabirwa n'ebyensula byali waggulu nnyo.*					
**430**	There are many transport options to get to the fistula centre/camp.<br>*Waliwo entambula yamilundi mingi okusobozesa okutuuka ewajjanjabilwa okutonya omusulo.*					
**431**	The repair facility was too far.<br>*Ekifo awajjanjabirwa kyaali wala nnyo.*					
**432**	The road conditions were bad.<br>*Embeera y'enguudo yali mbi nnyo*					
**433**	I fear traveling to the health facility because of pain and discomfort during travel.<br>*Ntya okugenda ku ddwaliro olw'obulumi n'obutawulira bulungi nga ntambula*					
**434**	I felt embarrassed about smell/leaking while traveling to the facility.<br>*Nawulira okuswala olw'ekivundu oba okutonnya nga ngenda ku ddwaliro*					
**435**	Repair facilities have enough doctors and nurses.<br>*Ebifo awajjanjabirwa birina basawo (ba dokita oba na nansi) abamala.*					
**436**	I worried that there are not many female health workers at the facility.<br>*Neeralikirira nti kuddwaliro teri basawo bakazi bamala.*					
**437**	Facilities do not have electricity, equipment, and/or supplies to provide health services.<br>*Ebifo awajjanjabirwa tebirina masannyalaze, ebyuuma ebikozesebwa n'ebikozesebwa ebirala okusobola okujjanjaba.*					

Under formatting review

**438**	I was afraid of harsh treatment by providers at the fistula centre/camp.<br>*Natya nti abasawo bajja kumpisa obubi mu kiffo ewajjajabibwa okutonya.*					
**439**	I did not want to stay for a long time at the fistula centre/camp to receive care.<br>*Ssaayagala kumala budde bungi mukifo ewajjanjabirwa oku tonnya..*					
**440**	I was told by health providers (in the past) that my fistula will heal itself.<br>*Abasawo bangamba emabegako nti ekirwadde ky'okutonnya kigya kuwona ky'okka.*					
**441**	I was told by health providers (in the past) that my fistula cannot be treated.<br>*Abasawo bangamba emabegako nti ekirwadde ky'okutonnya teki sobola kujajabibwa.*					
**442**	When I went to other facilities for a different health condition, doctors and nurses did not treat me properly.<br>*Bwennagenda ku mawaliro amalala olwe'ebeera endala, abasawo (ba dokita ne ba nansi) tebampisa bulungi*					
**443**	Repair /treatments I had in the past did not work. *Obujjanjabi bwenafuna emabegako tebwakola.*<br><br>[ask only if woman has had repair in the past]					
**444**	I depend on my mother-in-law for permission or money to seek care.<br>*Nnina okufuna olukusa oba ssente okuva ewa nyazaala wange abasajja okugenda okunnonya obujjanjabi.*					

[Facility code_Interviwer Code_Interview Number]

Questionnaire Number*:* ☐☐☐☐☐☐

SECTION 5: OPEN-ENDED QUESTIONS

Now we would like to hear **in your own words** about the barriers you faced and your experience of repair. [TURN ON RECORDER]

*Kati twagala kuwulira **mu bigambo byo** ku bikwata ku miziziko gyewasanga nebyoyiseemu ng'ojjanjabwa.*

501. a. Describe any other barriers you faced in getting to the fistula center for repair? [Probe: cultural, religion, gender, social norms, family, poverty, stigma]

Miziziko ki emirala gye wasanga mu kugenda mu kifo awajjanjabirwa ekirwadde ky'okutonnya?

b. Any barriers you faced after reaching the facility? [Probe: nurses, doctors, other health officers, anything else]

Buzibuki bwe wasanga bwewatuuka mu kifo awajjanjabirwa?

502. How did you get information about the service center? [Probe: from who, where]

Wamanya otya ebikwata ki kifo awajjanjabirwa?

503. Who are the people who helped you to get to the facility? [Probe: family member, friend, community-based agent, primary health center worker, other]

B'ani abakuyamba okutuuka ku kifo kino?

Probe: Ab'oluganda, ab'emikwano, omuweereza w'okukyalo, mukozi w'okuddwaliro, omulala)

504. Did you receive a referral to this fistula center? If so, please describe referral process.

Under formatting review

Eddwaliro eddala ly'akuweereza ku kifo kino awajjanjabirwa ekirwadde ky'okutonnya (fistula)? Bwekiba bwekityo, nnyonnyola engeri gyebaakuweerezaamu.

505. Describe how you came to the fistula center? [Probe, follow up on anything said early, confirm]

Watambudde/watambula otya okutuuka ku kifo kino awajjanjabirwa ekirwadde ky'okutonnya (fistula)?

506. Who paid for your transportation to this fistula center? [Probe: Myself, Family member or accompanying person, Fistula center staff, other]

Ani eyasasudde ebisale byo eby'entabula eyakutuusizza ku kifo kino awajjanjabirwa ekirwadde ky'okutonnya (fistula)?
(Probe: Nze nzennyini, ow'oluganda, oba omuntu awerekeddeko, mukozi w'ekifo awajjanjabirwa ekirwadde ky'okutonnya, omulala)

507. How is your relationship with your husband/intimate partner? [Probe: interactions, positive experience, negative experience, how it makes you feel]

Enkolaganayo ne balo eli'etya?

508. Now that your fistula has been repaired, do you think that your relationship with your husband/intimate partner will change? How? [Probe]

Kati engeri ekirwadde kyo eky'okutonnya gyekijjanjabiddwa, olowwoza enkolaganaayo ne balo eneekyuuka? Etya?

509. How is your relationship with your family and friends? [Probe: interactions, positive experience, negative experience, how it makes you feel]

Ekwataganayo eri abomumakago ne mikwano gyo eri etya?

510. Do you think your relationship with family and friends will change now that you are repaired? How? [Probe: social, relationships, interactions]

Osubira ekwataganayo na aboomumakago ne mikwano gyo enekyukako engeri gyofunye obujjanjabi? Enekyuka etya?

511. Do you know any other person having similar symptoms as you?

Omanyiiyo omuntu omulala yenna alina obubonero ng'obubwo?

512. What do you feel would be a good way for a person with your symptoms to get information about services for treatment? [Probe: from who, how, mode of communication, outreach]

Mukulabakwo, ngeri ki ennungi omuntu ari mubeera nga gyoyiseemu gyayinza okufunamu amawuliire assgakwata ndabirira nobujjanjabi bwe kilwadde kino?

Do you have any other comments for us at this time?

Olinayo byewandyagadde okwogerako kati?

Thank you for answering these questions for us.

Weebale nnyo okutuddiramu ebibuuzo bino.
