# Supplementary material for: Measuring barriers to fistula care: investigating composite measures for targeted fistula programming in Nigeria and Uganda
Source: BMC Womens Health. 2021 Apr 7;21:142. doi: 10.1186/s12905-021-01288-3 (PMC8028827; doi:10.1186/s12905-021-01288-3)
Supplement: Supplementary file 2 — Additional file 2: Supplemental Table 1. Distribution of barrier items. Supplemental Table 2. Quantitative measures summary. [file 12905_2021_1288_MOESM2_ESM.docx]

| Supplemental Table 1. Distribution of barrier items | | | | | |
| --- | --- | --- | --- | --- | --- |
|  | | Exploratory  n=315 | | Confirmatory  n=142 | |
|  |  | n | % | n | % |
| 1 | I felt ashamed of having fistula. |  |  |  |  |
|  | Strongly disagree | 10 | 3.2 | 1 | 0.7 |
|  | Disagree | 25 | 7.9 | 5 | 3.5 |
|  | Agree | 81 | 25.7 | 29 | 20.4 |
|  | Strongly agree | 197 | 62.5 | 107 | 75.4 |
|  | Missing | 2 | 0.6 | 0 | 0.0 |
| 2 | I felt depressed and anxious. |  |  |  |  |
|  | Strongly disagree | 3 | 1.0 | 0 | 0.0 |
|  | Disagree | 16 | 5.1 | 2 | 1.4 |
|  | Agree | 102 | 32.4 | 22 | 15.5 |
|  | Strongly agree | 190 | 60.3 | 117 | 82.4 |
|  | Missing | 4 | 1.3 | 1 | 0.7 |
| 3 | I felt worthless. |  |  |  |  |
|  | Strongly disagree | 14 | 4.4 | 2 | 1.4 |
|  | Disagree | 53 | 16.8 | 25 | 17.6 |
|  | Agree | 90 | 28.6 | 35 | 24.6 |
|  | Strongly agree | 155 | 49.2 | 79 | 55.6 |
|  | Missing | 3 | 1.0 | 1 | 0.7 |
| 4 | I felt guilty because I had fistula. |  |  |  |  |
|  | Strongly disagree | 36 | 11.4 | 16 | 11.3 |
|  | Disagree | 75 | 23.8 | 47 | 33.1 |
|  | Agree | 73 | 23.2 | 20 | 14.1 |
|  | Strongly agree | 128 | 40.6 | 58 | 40.8 |
|  | Missing | 3 | 1.0 | 1 | 0.7 |
| 5 | I felt I am not as complete as a person because I had fistula. |  |  |  |  |
|  | Strongly disagree | 11 | 3.5 | 1 | 0.7 |
|  | Disagree | 54 | 17.1 | 6 | 4.2 |
|  | Agree | 82 | 26.0 | 43 | 30.3 |
|  | Strongly agree | 163 | 51.7 | 91 | 64.1 |
|  | Missing | 5 | 1.6 | 1 | 0.7 |
| 6 | Having fistula made me feel unclean. |  |  |  |  |
|  | Strongly disagree | 18 | 5.7 | 3 | 2.1 |
|  | Disagree | 43 | 13.7 | 9 | 6.3 |
|  | Agree | 97 | 30.8 | 24 | 16.9 |
|  | Strongly agree | 153 | 48.6 | 105 | 73.9 |
|  | Missing | 4 | 1.3 | 1 | 0.7 |
| 7 | I depend on my husband or other male relatives for permission or money to seek care. |  |  |  |  |
|  | Strongly disagree | 63 | 20.0 | 13 | 9.2 |
|  | Disagree | 30 | 9.5 | 28 | 19.7 |
|  | Agree | 105 | 33.3 | 49 | 34.5 |
|  | Strongly agree | 114 | 36.2 | 49 | 34.5 |
|  | Missing | 3 | 1.0 | 3 | 2.1 |
| 8 | I had family obligations that kept me from coming to the fistula center. |  |  |  |  |
|  | Strongly disagree | 159 | 50.5 | 24 | 16.9 |
|  | Disagree | 95 | 30.2 | 75 | 52.8 |
|  | Agree | 27 | 8.6 | 23 | 16.2 |
|  | Strongly agree | 31 | 9.8 | 19 | 13.4 |
|  | Missing | 3 | 1.0 | 1 | 0.7 |
| 9 | I prefer to use traditional medicine. |  |  |  |  |
|  | Strongly disagree | 196 | 62.2 | 43 | 30.3 |
|  | Disagree | 79 | 25.1 | 73 | 51.4 |
|  | Agree | 33 | 10.5 | 18 | 12.7 |
|  | Strongly agree | 7 | 2.2 | 7 | 4.9 |
|  | Missing | 3 | 1.0 | 1 | 0.7 |
| 10 | I fear and/or dislike hospitals and medical clinics. |  |  |  |  |
|  | Strongly disagree | 179 | 56.8 | 32 | 22.5 |
|  | Disagree | 105 | 33.3 | 81 | 57.0 |
|  | Agree | 18 | 5.7 | 18 | 12.7 |
|  | Strongly agree | 10 | 3.2 | 10 | 7.0 |
|  | Missing | 3 | 1.0 | 1 | 0.7 |
| 11 | I felt embarrassed because of my condition. |  |  |  |  |
|  | Strongly disagree | 15 | 4.8 | 4 | 2.8 |
|  | Disagree | 40 | 12.7 | 10 | 7.0 |
|  | Agree | 99 | 31.4 | 33 | 23.2 |
|  | Strongly agree | 158 | 50.2 | 94 | 66.2 |
|  | Missing | 3 | 1.0 | 1 | 0.7 |
| 12 | I felt isolated because of my fistula condition. |  |  |  |  |
|  | Strongly disagree | 42 | 13.3 | 4 | 2.8 |
|  | Disagree | 60 | 19.0 | 28 | 19.7 |
|  | Agree | 83 | 26.3 | 37 | 26.1 |
|  | Strongly agree | 124 | 39.4 | 72 | 50.7 |
|  | Missing | 6 | 1.9 | 1 | 0.7 |
| 13 | People who knew I had fistula avoided me. |  |  |  |  |
|  | Strongly disagree | 71 | 22.5 | 12 | 8.5 |
|  | Disagree | 74 | 23.5 | 53 | 37.3 |
|  | Agree | 73 | 23.2 | 26 | 18.3 |
|  | Strongly agree | 94 | 29.8 | 50 | 35.2 |
|  | Missing | 3 | 1.0 | 1 | 0.7 |
| 14 | Women with fistula are treated like outcasts. |  |  |  |  |
|  | Strongly disagree | 27 | 8.6 | 8 | 5.6 |
|  | Disagree | 45 | 14.3 | 25 | 17.6 |
|  | Agree | 97 | 30.8 | 35 | 24.6 |
|  | Strongly agree | 143 | 45.4 | 73 | 51.4 |
|  | Missing | 3 | 1.0 | 1 | 0.7 |
| 15 | My husband/intimate partner treated me poorly initially. |  |  |  |  |
|  | Strongly disagree | 134 | 42.5 | 28 | 19.7 |
|  | Disagree | 85 | 27.0 | 68 | 47.9 |
|  | Agree | 38 | 12.1 | 9 | 6.3 |
|  | Strongly agree | 52 | 16.5 | 33 | 23.2 |
|  | Missing | 6 | 1.9 | 4 | 2.8 |
| 16 | My husband/intimate partner treated me poorly later on. |  |  |  |  |
|  | Strongly disagree | 119 | 37.8 | 32 | 22.5 |
|  | Disagree | 94 | 29.8 | 60 | 42.3 |
|  | Agree | 37 | 11.7 | 14 | 9.9 |
|  | Strongly agree | 60 | 19.0 | 33 | 23.2 |
|  | Missing | 5 | 1.6 | 3 | 2.1 |
| 17 | My husband/intimate partner abandoned me. |  |  |  |  |
|  | Strongly disagree | 153 | 48.6 | 30 | 21.1 |
|  | Disagree | 73 | 23.2 | 65 | 45.8 |
|  | Agree | 25 | 7.9 | 7 | 4.9 |
|  | Strongly agree | 57 | 18.1 | 37 | 26.1 |
|  | Missing | 7 | 2.2 | 3 | 2.1 |
| 18 | I did not have someone to care for me and help me manage my condition at home. |  |  |  |  |
|  | Strongly disagree | 113 | 35.9 | 21 | 14.8 |
|  | Disagree | 113 | 35.9 | 76 | 53.5 |
|  | Agree | 40 | 12.7 | 11 | 7.7 |
|  | Strongly agree | 46 | 14.6 | 33 | 23.2 |
|  | Missing | 3 | 1.0 | 1 | 0.7 |
| 19 | I did not have someone to support me in seeking and reaching care at the fistula center. |  |  |  |  |
|  | Strongly disagree | 114 | 36.2 | 22 | 15.5 |
|  | Disagree | 102 | 32.4 | 77 | 54.2 |
|  | Agree | 41 | 13.0 | 16 | 11.3 |
|  | Strongly agree | 54 | 17.1 | 26 | 18.3 |
|  | Missing | 4 | 1.3 | 1 | 0.7 |
| 20 | I was told by other people that my fistula would heal itself. |  |  |  |  |
|  | Strongly disagree | 148 | 47.0 | 28 | 19.7 |
|  | Disagree | 81 | 25.7 | 72 | 50.7 |
|  | Agree | 51 | 16.2 | 24 | 16.9 |
|  | Strongly agree | 32 | 10.2 | 17 | 12.0 |
|  | Missing | 3 | 1.0 | 1 | 0.7 |
| 21 | I did not know that fistula is a medical condition that can be treated. |  |  |  |  |
|  | Strongly disagree | 60 | 19.0 | 17 | 12.0 |
|  | Disagree | 71 | 22.5 | 58 | 40.8 |
|  | Agree | 61 | 19.4 | 17 | 12.0 |
|  | Strongly agree | 120 | 38.1 | 49 | 34.5 |
|  | Missing | 3 | 1.0 | 1 | 0.7 |
| 22 | I believed that having OF was a curse. |  |  |  |  |
|  | Strongly disagree | 114 | 36.2 | 47 | 33.1 |
|  | Disagree | 93 | 29.5 | 32 | 22.5 |
|  | Agree | 30 | 9.5 | 11 | 7.7 |
|  | Strongly agree | 75 | 23.8 | 51 | 35.9 |
|  | Missing | 3 | 1.0 | 1 | 0.7 |
| 23 | I believed that my OF was caused by diabolic means. |  |  |  |  |
|  | Strongly disagree | 120 | 38.1 | 42 | 29.6 |
|  | Disagree | 70 | 22.2 | 46 | 32.4 |
|  | Agree | 43 | 13.7 | 13 | 9.2 |
|  | Strongly agree | 79 | 25.1 | 40 | 28.2 |
|  | Missing | 3 | 1.0 | 1 | 0.7 |
| 24 | I did not know where to go for fistula repair. |  |  |  |  |
|  | Strongly disagree | 38 | 12.1 | 6 | 4.2 |
|  | Disagree | 48 | 15.2 | 50 | 35.2 |
|  | Agree | 87 | 27.6 | 21 | 14.8 |
|  | Strongly agree | 136 | 43.2 | 64 | 45.1 |
|  | Missing | 6 | 1.9 | 1 | 0.7 |
| 25 | I did not have money to pay for medical care to treat my fistula. |  |  |  |  |
|  | Strongly disagree | 19 | 6.0 | 6 | 4.2 |
|  | Disagree | 63 | 20.0 | 37 | 26.1 |
|  | Agree | 88 | 27.9 | 21 | 14.8 |
|  | Strongly agree | 141 | 44.8 | 77 | 54.2 |
|  | Missing | 4 | 1.3 | 1 | 0.7 |
| 26 | I was unable to work because of the discomfort caused by my fistula condition. |  |  |  |  |
|  | Strongly disagree | 23 | 7.3 | 7 | 4.9 |
|  | Disagree | 48 | 15.2 | 24 | 16.9 |
|  | Agree | 91 | 28.9 | 48 | 33.8 |
|  | Strongly agree | 148 | 47.0 | 61 | 43.0 |
|  | Missing | 5 | 1.6 | 2 | 1.4 |
| 27 | I was unable to work because of stigma associated with my fistula condition. |  |  |  |  |
|  | Strongly disagree | 54 | 17.1 | 6 | 4.2 |
|  | Disagree | 71 | 22.5 | 46 | 32.4 |
|  | Agree | 78 | 24.8 | 40 | 28.2 |
|  | Strongly agree | 108 | 34.3 | 47 | 33.1 |
|  | Missing | 4 | 1.3 | 3 | 2.1 |
| 28 | Once others learnt of my condition, they did not allow me to work/earn money. |  |  |  |  |
|  | Strongly disagree | 66 | 21.0 | 11 | 7.7 |
|  | Disagree | 66 | 21.0 | 44 | 31.0 |
|  | Agree | 96 | 30.5 | 48 | 33.8 |
|  | Strongly agree | 81 | 25.7 | 37 | 26.1 |
|  | Missing | 6 | 1.9 | 2 | 1.4 |
| 29 | The cost of transportation to repair sites and accommodation was too high. |  |  |  |  |
|  | Strongly disagree | 38 | 12.1 | 8 | 5.6 |
|  | Disagree | 85 | 27.0 | 23 | 16.2 |
|  | Agree | 83 | 26.3 | 55 | 38.7 |
|  | Strongly agree | 104 | 33.0 | 55 | 38.7 |
|  | Missing | 5 | 1.6 | 1 | 0.7 |
| 30 | There are many transport options to get to the fistula center. |  |  |  |  |
|  | Strongly agree | 107 | 34.0 | 67 | 47.2 |
|  | Agree | 95 | 30.2 | 46 | 32.4 |
|  | Disagree | 78 | 24.8 | 23 | 16.2 |
|  | Strongly disagree | 29 | 9.2 | 5 | 3.5 |
|  | Missing | 6 | 1.9 | 1 | 0.7 |
| 31 | The repair facility was too far. |  |  |  |  |
|  | Strongly disagree | 28 | 8.9 | 4 | 2.8 |
|  | Disagree | 77 | 24.4 | 26 | 18.3 |
|  | Agree | 84 | 26.7 | 50 | 35.2 |
|  | Strongly agree | 121 | 38.4 | 61 | 43.0 |
|  | Missing | 5 | 1.6 | 1 | 0.7 |
| 32 | The road conditions were bad. |  |  |  |  |
|  | Strongly disagree | 62 | 19.7 | 12 | 8.5 |
|  | Disagree | 102 | 32.4 | 45 | 31.7 |
|  | Agree | 83 | 26.3 | 47 | 33.1 |
|  | Strongly agree | 64 | 20.3 | 37 | 26.1 |
|  | Missing | 4 | 1.3 | 1 | 0.7 |
| 33 | I fear traveling to the health facility because of pain and discomfort during travel. |  |  |  |  |
|  | Strongly disagree | 81 | 25.7 | 14 | 9.9 |
|  | Disagree | 82 | 26.0 | 63 | 44.4 |
|  | Agree | 79 | 25.1 | 31 | 21.8 |
|  | Strongly agree | 68 | 21.6 | 33 | 23.2 |
|  | Missing | 5 | 1.6 | 1 | 0.7 |
| 34 | I felt embarrassed about smell/leaking while traveling to the facility. |  |  |  |  |
|  | Strongly disagree | 40 | 12.7 | 4 | 2.8 |
|  | Disagree | 34 | 10.8 | 23 | 16.2 |
|  | Agree | 96 | 30.5 | 44 | 31.0 |
|  | Strongly agree | 140 | 44.4 | 70 | 49.3 |
|  | Missing | 5 | 1.6 | 1 | 0.7 |
| 35 | Repair facilities have enough doctors and nurses. |  |  |  |  |
|  | Strongly agree | 213 | 67.6 | 102 | 71.8 |
|  | Agree | 75 | 23.8 | 28 | 19.7 |
|  | Disagree | 14 | 4.4 | 9 | 6.3 |
|  | Strongly disagree | 7 | 2.2 | 2 | 1.4 |
|  | Missing | 6 | 1.9 | 1 | 0.7 |
| 36 | I worried that there are not many female health workers at the facility. |  |  |  |  |
|  | Strongly disagree | 149 | 47.3 | 38 | 26.8 |
|  | Disagree | 91 | 28.9 | 77 | 54.2 |
|  | Agree | 35 | 11.1 | 12 | 8.5 |
|  | Strongly agree | 35 | 11.1 | 14 | 9.9 |
|  | Missing | 5 | 1.6 | 1 | 0.7 |
| 37 | Facilities do not have electricity, equipment, and/or supplies to provide health services. |  |  |  |  |
|  | Strongly disagree | 200 | 63.5 | 45 | 31.7 |
|  | Disagree | 75 | 23.8 | 83 | 58.5 |
|  | Agree | 16 | 5.1 | 4 | 2.8 |
|  | Strongly agree | 20 | 6.3 | 9 | 6.3 |
|  | Missing | 4 | 1.3 | 1 | 0.7 |
| 38 | I was afraid of harsh treatment by providers at the fistula center. |  |  |  |  |
|  | Strongly disagree | 102 | 32.4 | 24 | 16.9 |
|  | Disagree | 92 | 29.2 | 79 | 55.6 |
|  | Agree | 64 | 20.3 | 24 | 16.9 |
|  | Strongly agree | 53 | 16.8 | 14 | 9.9 |
|  | Missing | 4 | 1.3 | 1 | 0.7 |
| 39 | I did not want to stay for a long time at the fistula center to receive care. |  |  |  |  |
|  | Strongly disagree | 96 | 30.5 | 16 | 11.3 |
|  | Disagree | 70 | 22.2 | 72 | 50.7 |
|  | Agree | 90 | 28.6 | 34 | 23.9 |
|  | Strongly agree | 54 | 17.1 | 19 | 13.4 |
|  | Missing | 5 | 1.6 | 1 | 0.7 |
| 40 | I was told by health providers (in the past) that my fistula will heal itself. |  |  |  |  |
|  | Strongly disagree | 193 | 61.3 | 51 | 35.9 |
|  | Disagree | 80 | 25.4 | 72 | 50.7 |
|  | Agree | 22 | 7.0 | 8 | 5.6 |
|  | Strongly agree | 15 | 4.8 | 10 | 7.0 |
|  | Missing | 5 | 1.6 | 1 | 0.7 |
| 41 | I was told by health providers (in the past) that my fistula cannot be treated. |  |  |  |  |
|  | Strongly disagree | 191 | 60.6 | 61 | 43.0 |
|  | Disagree | 84 | 26.7 | 68 | 47.9 |
|  | Agree | 18 | 5.7 | 6 | 4.2 |
|  | Strongly agree | 16 | 5.1 | 6 | 4.2 |
|  | Missing | 6 | 1.9 | 1 | 0.7 |
| 42 | When I went to other facilities for a different health condition, doctors and nurses did not treat me properly. |  |  |  |  |
|  | Strongly disagree | 103 | 32.7 | 30 | 21.1 |
|  | Disagree | 87 | 27.6 | 76 | 53.5 |
|  | Agree | 63 | 20.0 | 16 | 11.3 |
|  | Strongly agree | 52 | 16.5 | 17 | 12.0 |
|  | Missing | 10 | 3.2 | 3 | 2.1 |
| 43 | Repair /treatments I had in the past did not work. |  |  |  |  |
|  | Strongly disagree | 85 | 27.0 | 28 | 19.7 |
|  | Disagree | 27 | 8.6 | 18 | 12.7 |
|  | Agree | 38 | 12.1 | 9 | 6.3 |
|  | Strongly agree | 99 | 31.4 | 15 | 10.6 |
|  | Missing | 66 | 21.0 | 72 | 50.7 |

Supplemental Table 2: Quantitative measures summary

| **Barriers to Fistula Care Index (17-item)** | | | |
| --- | --- | --- | --- |
| *Sub-scale* | Item | Response categories | |
| *Limited awareness*  *(4-item)* | I did not know that fistula is a medical condition that can be treated. | Strongly disagree  Disagree  Agree  Strongly agree | |
|  | I believed that having fistula was a curse. |  |  |
|  | I believed that my fistula was caused by diabolic means. |  |  |
|  | I did not know where to go for fistula repair. |  |  |
| *Social abandonment*  *(6-item)* | People who knew I had fistula avoided me. | Strongly disagree  Disagree  Agree  Strongly agree | |
|  | My husband/intimate partner treated me poorly initially. |  |  |
|  | My husband/intimate partner treated me poorly later on. |  |  |
|  | My husband/intimate partner abandoned me. |  |  |
|  | I did not have someone to care for me and help me manage my condition at home. |  |  |
|  | I did not have someone to support me in seeking and reaching care at the fistula center. |  |  |
| *Internalized stigma*  *(7-item)* | I felt ashamed of having fistula. | Strongly disagree  Disagree  Agree  Strongly agree | |
|  | I felt worthless. |  |  |
|  | I felt guilty because I had fistula. |  |  |
|  | I felt I am not as complete as a person because I had fistula. |  |  |
|  | Having fistula made me feel unclean. |  |  |
|  | I felt embarrassed because of my condition. |  |  |
|  | I felt isolated because of my fistula condition. |  |  |
| **Financial/Transport Inaccessibility Index (6-item)** | | | |
| Item | | Response categories | |
| I did not have money to pay for medical care to treat my fistula. | | Strongly disagree  Disagree  Agree  Strongly agree |  |
| I was unable to work because of stigma associated with my fistula condition. | |  |  |
| There are not enough transport options to get to the fistula center. | |  |  |
| The cost of transportation to repair sites and accommodation was too high. | |  |  |
| The repair facility was too far. | |  |  |
| The road conditions were bad. | |  |  |
